# Controlled Plasma Membrane Delivery of FGFR1 and Modulation of Signaling by a Novel Regulated Anterograde RTK Transport Pathway

**DOI:** 10.3390/cancers15245837

**Published:** 2023-12-14

**Authors:** Claire Leist Hinsch, Jagadish Kummetha Venkata, Tien Hsu, Vincent Dammai

**Affiliations:** 1 Hollings Cancer Center, Medical University of South Carolina, Charleston, SC 29401, USAjagadishk.in@mouritech.com (J.K.V.); 2 Department of Pathology and Laboratory Medicine, Medical University of South Carolina, Charleston, SC 29401, USA; 3 Graduate Institute of Biomedical Sciences, China Medical University, Taichung 40433, Taiwan; 4 Aldevron LLC (Danaher Corporation), Fargo, ND 58104, USA

**Keywords:** RART pathway, FGFR1, receptor trafficking, RTK signaling, solid tumors

## Abstract

**Simple Summary:**

Fibroblast Growth Factor Receptor-1 (FGFR1), a major receptor tyrosine kinase (RTK), regulates early embryonic development and organogenesis. However, overexpression of FGFR1 is seen in many human solid tumors and is associated with poor prognosis, resistance to multiple therapies and aggressive metastatic spread. Our aim was to study the mechanisms controlling FGFR1 activity, with an ultimate goal of identifying key therapeutic targets for solid tumors. We unexpectedly found that a major splice variant, FGFR1 IIIc, was sequestered in intracellular vesicles under normal conditions. We investigated this phenomenon in detail to identify the sequestration mechanism and further elucidated how the sequestration was released to permit receptor translocation to the plasma membrane (PM) in response to microenvironmental cues. The novel pathway, a first such mechanism identified for RTK, regulates PM localization of FGFR1 IIIc, thereby determining its signal output. This knowledge will aid in the development of next-generation strategies to silence FGFR1 in solid tumors.

**Abstract:**

How human FGFR1 localizes to the PM is unknown. Currently, it is assumed that newly synthesized FGFR1 is continuously delivered to the PM. However, evidence indicates that FGFR1 is mostly sequestered in intracellular post-Golgi vesicles (PGVs) under normal conditions. In this report, live-cell imaging and total internal reflection fluorescence microscopy (TIRFM) were employed to study the dynamics of these FGFR1-positive vesicles. We designed recombinant proteins to target different transport components to and from the FGFR1 vesicles. Mouse embryoid bodies (mEBs) were used as a 3D model system to confirm major findings. Briefly, we found that Rab2a, Rab6a, Rab8a, RalA and caveolins are integral components of FGFR1-positive vesicles, representing a novel compartment. While intracellular sequestration prevented FGFR1 activation, serum starvation and hypoxia stimulated PM localization of FGFR1. Under these conditions, FGFR1 C-terminus acts as a scaffold to assemble proteins to (i) inactivate Rab2a and release sequestration, and (ii) assemble Rab6a for localized activation of Rab8a and RalA-exocyst to deliver the receptor to the PM. This novel pathway is named Regulated Anterograde RTK Transport (RART). This is the first instance of RTK regulated through control of PM delivery.

## 1. Introduction

Receptor tyrosine kinases (RTKs) participate in a multitude of signaling pathways that influence normal development and growth [1]. Following ligand binding and activation, RTKs are immediately removed from the cell surface through endocytosis to prevent prolonged signaling [2,3], which is the universally accepted model for downregulation of RTK activity. In this model, however, the initial cell surface localization of RTKs is assumed to occur through continuous delivery of receptors from PGVs. Increased PM accumulation of RTKs is observed in many cancers and is widely known to cause tumor resistance to chemo- and radiation therapies by hyperactivating signaling pathways and promoting epithelial-to-mesenchymal transition (EMT), leading to metastatic spread.

Currently, there are no published studies addressing how FGFR1 localizes to the PM. Targeting of PM-localized proteins is a complex process influenced by multiple factors and is poorly understood. Cell polarity establishes apical and basolateral PM domains, allowing asymmetric distribution of PM proteins [4,5,6]. Studies involving ablation of vesicle compartments and trafficking of model substrates have identified diverse signals such as C-terminal domains, post-translational modifications, clustering and GPI-anchors that facilitate cargo sorting through vectoral or transcytotic routes [7,8,9,10,11]. In the case of FGFR1, it is simply assumed that newly synthesized FGFR1 follows a vectoral route; i.e., the receptor is continuously delivered to the PM after passage through Golgi. Consequently, increased *FGFR1* expression is presumed to accumulate the receptor on the PM, resulting in hyper-activation governed by low hopping rates [12,13] and density-dependent oligomerization [14,15]. However, indirect evidence has shown that serum starvation induces PM accumulation of FGFR1, suggesting the existence of regulatory steps in FGFR1 transport [14].

FGFR signaling in mammals is complex and involves 4 RTK-type FGF receptors 1–4, plus their multiple receptor isoforms, and 23 fibroblast growth factors (FGFs) [16]. FGFR1 is a major RTK, one of the earliest RTKs activated during development [17], and mouse *Fgfr1* knockouts exhibit early embryonic lethality [18,19,20]. Signaling through FGFR1 is utilized repeatedly in several critical developmental events such as directed cell migration, developmentally regulated EMT, cell differentiation, vascular development and branching morphogenesis [21,22,23,24,25,26]. Given that each FGF receptor is activated by more than one FGF ligand, it should be important to understand how signaling from the FGFR-FGF system is activated and precisely controlled to elicit cellular outcomes critical for organism development and disease progression. In this context, theoretical models such as the ‘threshold model’ propose distinct activation thresholds of different FGF receptors that fine-tune their responses to the extracellular ligands [27]. Other arguments favor FGF ligands directly controlling the quality of receptor activation through inducing unique ligand-dependent structural changes in the FGF receptor involved [28].

We had earlier noted predominantly intracellular vesicle localization of FGFR1, a feature that is conserved from *Drosophila* to humans [14,23]. Intracellular sequestration is a known mechanism for selective utilization of some cell surface receptors but there are no prior studies to indicate that RTKs are regulated in this manner. Prominent examples of sequestration of PM proteins in intracellular storage vesicles are glucose transporter 4 (GLUT4), α-amino-3-hydroxy-5-methyl-4-isoxazolepropionic acid (AMPA) receptor, epithelial sodium channels (ENACs) and aquaporin-2 (AQP-2) [29,30,31,32]. In these cases, regulated non-secretory exocytosis delivers the sequestered cargo to PM upon a stimulus. Regulated non-secretory exocytosis occurs through complete vesicle fusion within minutes—less than 10 min in the case of GLUT4—indicating fast-acting PM targeting mechanisms [33]. Recycling vesicles also deliver recycled receptors to the PM predominantly through full vesicle fusion [34], albeit at slower kinetics compared to non-secretary exocytosis.

We previously demonstrated FGFR1 endocytosis defects in human *von Hippel-Lindau* (*hVHL*) mutant kidney cancer cells, resulting in excessive accumulation of FGFR1 on the PM and increased signaling activity [14]. In follow-up studies, we noted FGFR1 IIIc localization in the intracellular vesicles. Multiple alternative splice forms of mammalian FGFR1-3 exhibit distinct spatial expression patterns throughout the body. The IIIb isoforms of FGFR1-3 generally are expressed in epithelia, while IIIc isoforms of FGFR1-3 are expressed in the mesenchyme, and their localized activities are critical in organogenesis. FGFR1 IIIb and FGFR1 IIIc are prominent FGFR1 splice variants differing in the inclusion of exon 8 or 9, respectively, in the IgIII loop. While deletion of exon 9, resulting in FGFR1 IIIc knockout, is embryonic lethal, deletion of exon 8 (FGFR1 IIIb knockout) has no overt phenotypes in mouse [35]. FGFR1 IIIc is highly expressed in TGF-β1-induced EMT of murine NMuMG epithelial mammary gland cells [36]. Although the expression of specific FGFR1 isoforms is generally overlooked in most published cancer studies, human pancreatic and prostate cancers have been reported to overexpress FGFR1 IIIc [37,38]. FGFR1 IIIc expression is upregulated at the transcriptional level by FOXC1 [39], an important transcription factor required during regulated EMT events in organ development. On the other hand, the ZEB1/2 transcription factors, which also play a role in EMT, influence isoform switching to FGFR1 IIIc via regulating alternative splicing of FGFR1 mRNA [40]. These studies demonstrated multiple levels of regulation of FGFR1 IIIc expression. In this report, we have identified a novel Regulated Anterograde RTK Transport (RART) pathway that controls the cell surface levels of FGFR1 IIIc. We first show that the receptor is normally sequestered in intracellular vesicles to prevent signaling. We characterized the sequestered compartment and identified its molecular components. We next identified the molecular mechanisms activated to allow for PM localization of the receptor in response to microenvironmental cues. Our results show that the RART pathway constitutes a novel mechanism to regulate FGFR1 signaling through control of FGFR1 cell surface localization. This is the first example of RTK signaling regulated at the post-Golgi to PM transport level.

## 2. Materials and Methods

### 2.1. Cell Culture

Most cell lines were purchased from American Type Culture Collection (ATCC, Manassas, VA, USA) and cultured in normoxia (Nx; 21% O_2_) or hypoxia (Hx; 1.5% O_2_) in standard ATCC-specified DMEM growth medium at 37 °C with 5% CO_2_ and 100 units/mL penicillin and 0.1 mg/mL streptomycin. The SK-RC-31 cell line was generated by Memorial Sloan Kettering Cancer Center (New York, NY, USA) from metastatic tumor developed at lung site of a patient with *VHL*-deficient renal cell carcinoma (RCC), and was a gift from Dr. Andrew Kraft (Hollings Cancer Center). Cell culture ware, media and supplements were purchased from Thermo Fisher Scientific (Waltham, MA, USA). Cells were briefly trypsinized (Trypsin-EDTA 0.25%, Cat # 25200114, GIBCO, Thermo Fisher Scientific) to retain small cell clusters and seeded at ~30% density on fibronectin-coated 100 mm (Cat # 354451, Corning, Glendale, AZ, USA) or 60 mm culture dishes (Cat # 354403, Corning) to allow cell growth in clusters. At 60% confluency, complete medium was removed, cells rinsed once with PBS and fresh medium added to begin serum starvation or Hx treatments. For serum starvation (SS), cells were cultured in FBS-free minimal medium/low glucose (Cat # 11885084, GIBCO, Thermo Fisher Scientific) and grown on fibronectin-coated 100 mm or 60 mm dishes, or on 35 mm MatTek glass bottom dishes (Part # p35G-1.5-14-C) coated with fibronectin for live cell imaging. Fibronectin coating of 35 mm MatTek glass bottom dishes was freshly conducted at room temperature, covering the glass bottom area for 90 min with fibronectin diluted in Ca^2+^/Mg^2+^-free DPBS (Cat # 14190144, GIBCO, Thermo Fisher Scientific) at 2.5 μg/cm^2^ using fibronectin isolated from human plasma (Cat # 5050, Advanced Biomatrix, Carlsbad, CA, USA), followed by removing the fibronectin solution and air-drying the coated dishes for 45 min at room temperature in laminar hood. For Hx treatment, complete medium was used and cells were similarly seeded on fibronectin-coated 100 mm or 60 mm cell culture dishes, or 35 mm MatTek glass bottom dishes coated with fibronectin, and maintained at 1.5% O_2_ in HERAcell 150i (Cat # 51026408, Thermo Fisher Scientific) CO_2_ incubator with IR sensor and 1–21% O_2_ controller. Cell counts, for routine cell passaging and all experimental setups, were obtained using automated cell counter Nexcelom Cellometer (SKU # Cellometer Auto T4 plus, Nexcelom, PerkinElmer, Waltham, MA, USA). Trypan blue exclusion was used to acquire data on live/dead cell ratio (viability index) using Nexcelom Cellometer.

### 2.2. Cell Transfection

Stable and transient transfection of various recombinant plasmids were carried out using Amaxa Nucleofector 2 (Cat # AAB 1001, Lonza, Rockland, ME, USA) or FuGENE HD (Cat # HD-5000, FuGENE, Middleton, WI, USA). Nucleofector Kit’s instructions were followed to minimize cell death and maximize efficiency. Based on the result, Kit V reagents (Cat # VCA 1003) and A23 program were chosen for all Nucleofector-based transfection. For small interfering RNA (siRNA) and mouse embryoid body (mEB) experiments, following 12 h of transfection of siRNA or recombinant plasmids mixed in opti-MEM (Cat # 31985062 GIBCO) with FuGENE HD reagent, complete medium was added and cells were allowed to recover for 12 h and subsequently incubated with respective medium (see above) to initiate Nx, SS or Hx treatment for indicated durations for siRNA assays, and murine embryonic stem cells (mESCs) were placed in hanging drops and mEB formation steps were followed as described below.

### 2.3. ES Cell Culture and Differentiation

mESC culture and mEB differentiation was performed as described [41] with slight modifications. Briefly, mESCs were counted using Nexcelom Cellometer and cells were resuspended in complete media deprived of Leukemia Inhibitory Factor (LIF) and cultured in 30 µL hanging drops (600 cells/drop) for 2 days. Aggregates were formed in the hanging drops and subsequently the hanging drops were collected and pooled into suspension culture (day 0). On day 7 of suspension culture, individual mEBs were carefully picked with autopipette tips (cut with a sterile blade to widen the tip) and plated on 0.1% gelatin-coated 24-well tissue culture plate. The percentage of mEBs showing spontaneous beating was counted up to day 14. For transfection, mESCs were transfected with recombinant plasmids using FuGENE HD following manufacturer‘s instructions. Transfected mESCs were allowed to recover in complete media for 12 h before being placed in hanging drops in defined media. The mEBs that began to beat were marked with color-coded ink at the bottom of the plate, to follow their beating and to avoid repeat counting. In this manner, the number of mEBs beating each day in each experimental condition was recorded from day 7 to day 14. Pharmacological inhibitor treatments were initiated, where needed, on day 0 of mEB suspension culture. At day 0 of suspension culture, cells were subjected to Nx, SS or Hx until conclusion of experimentation on day 14.

### 2.4. Subcellular Fractionation

An amount of 3.6 × 10^7^ cells were washed with PBS and resuspended in ice-cold fractionation buffer (0.25 M sucrose, 0.05 M Tris-HCl (pH 7.4), 0.01 M KCl, 0.02 M MgCl_2_, 0.02 M Na-EDTA, 0.01 M β-mercaptoethanol) with protease inhibitor cocktail (SKU # 1183615300, Complete Mini Protease Inhibitor Cocktail, Roche Diagnostics GmbH, Mannheim, Germany). Cells in 2.0 mL ice-cold fractionation buffer were homogenized with 2.0 mL KIMBLE KONTES Dounce homogenizer (Cat # 885300-0002, Kontes, Vineland, NJ, USA) using 45 strokes with tight-fitting pestle to rupture cells. Nuclei and PM were separated by microcentrifugation in refrigerated Sorvall Legend Micro 17R microcentrifuge (Cat # 75002440, max 17,000× *g*, Thermo Fisher Scientific) fitted with fixed angle rotor at 3000× *g* for 10 min. Large, intact organelles were pelleted from post-nuclear supernatant also by microcentrifugation at 10,000× *g* for 10 min. Subsequently, differential ultracentrifugation was performed at 20,000× *g* for 20 min, 50,000× *g* for 60 min and 100,000× *g* for 60 min. Differential ultracentrifugations were performed in balanced open-top 1.4 mL (11 × 34 mm) thick-walled polycarbonate tubes using TLS-55 swinging rotor in Beckman Coulter Optima TLX-120 ultracentrifuge.

### 2.5. Microscopy

Fixed and live cell imaging was performed using Olympus FluoView FV300 (IX70 stand) and Zeiss LSM 510 META (Axiovert 200M stand) inverted confocal fluorescence imaging microscopes, and images were acquired using software FluoView 300 V5.0 (scanning speed 4 frames/s at 512 × 512 pixels) and LSM 510 V4.2 (scanning speed 5 frames/s at 512 × 512 pixels), respectively. Live cell imaging was performed on unenclosed microscope stage using NEVTEK ASI 400 airstream incubator (NEVTEK, Williamsville, VA, USA) to control temperature at 37+/−0.2 °C and with cells grown on fibronectin-coated 35 mm MatTek glass bottom dishes, after replacing medium with pre-warmed CO_2_-independent medium (Cat # 18045088, GIBCO, Thermo Fisher Scientific) for longer-duration imaging (up to 20 min) without needing microscope environmental chamber. To pre-warm the stage and objectives, NEVTEK ASI 400 airstream incubator was initiated 10 min prior to placing cells grown on 35 mm glass bottom dishes on the microscope stage and imaging was initiated 5 min later to allow temperature equilibration and avoid thermal drift. Confocal image scan was taken every 3.2 s for a total duration of 5 min for live cell imaging. Specific time points of observation are indicated in the figures. Co-localization was assessed by frame-by-frame replay of time-lapse videos from live cell imaging and further confirmed by re-imaging the same samples after immediately fixing with 2% paraformaldehyde + PBS for 5 min. Fluorescence intensity from total internal reflection fluorescence microscopy (TIRFM) images was measured using NIH ImageJ software version 1.48 (see below).

Indirect immunofluorescence was performed as described previously [14,23] with cells grown on fibronectin coated 1.5 mm thick 10 mm glass coverslips (Cat # NC1272767, Thermo Fisher Scientific) placed in 12-well plates. Fibronectin coating of coverslips was essentially as described above, except that the glass coverslips were pre-sterilized in 100% ethanol and air-dried before placing them onto 20 μL drops of freshly diluted fibronectin solution (see above) in 100 mm culture dish for 40 min in laminar hood and inverting them onto fresh 100 mm culture dish to air-dry the coated side for 45 min before placing the coverslips in 12-well plates and seeding the cells. All images were lightly adjusted for brightness/contrast levels uniformly across all pixels using Adobe Photoshop version CS5, and no other image manipulations were made.

### 2.6. Total Internal Reflection Fluorescence Microscopy (TIRFM)

Cells at 80% confluency in 35 mm MatTek glass bottom dishes were mounted in a Tokai Hit warming stage on a Nikon Ti live cell microscope equipped for TIRFM. The system was aligned and 5 stage positions were chosen for imaging. Images were collected at 200 ms exposure with 15 s intervals using a 60× objective, and a Photometrics Coolsnap HQ2 camera and processed using Nikon Elements software. Total TIRFM imaging duration was 5–15 min. Nx, SS and Hx treatment were as described above. Live video segments of TIRFM results were studied and video segments showing PM fusion events were analyzed for fluorescence intensity using NIH ImageJ version 1.48.

### 2.7. Quantification of Microscopic Images

Each experiment was performed 5 times (n = 5). A total of 100 cells were examined for each experiment and greater than 75% of cells exhibiting the same pattern (co-localization with FGFR1-containing vesicles and FGFR1 PM localization) were scored as positive. The extent of co-localization was scored as + to ++++, with ++++ being >95%. In addition, from the above 100 cells scored as positive, 200 individual vesicles were counted per cell (total of 10 cells counted) and vesicle co-localization was expressed as % +/− SD in numerical value.

### 2.8. Western Blotting and Immunofluorescence

Antibodies against EGFR (Cat # 4267), phospho-FGFR1 (Cat # 52928), AKT (Cat # 9272), pAKT-Ser473 (Cat # 4058, 193H12), Erk1/2 (Cat # 9102) and phospho-Erk1/2 Thr202/Tyr204 (Cat # 4377), and rabbit monoclonal FGFR1-Ab3 (Cat # 9740) that detects the C-terminal intracellular domain were from Cell Signaling Technology (Danvers, MA, USA). Unless indicated otherwise, FGFR1-Ab3 was used to detect R1-GFP and ΔCR1-GFP in all Western blots. Rabbit polyclonal anti-RalA (Cat # 67093-1-Ig) and anti-RalB (Cat # 67094-1-Ig) antibodies were from ProteinTech Group (Rosemont, IL, USA). Mouse monoclonal antibodies against Rab8a (Cat # WH0004218M2), β-actin (Cat # A5441), β-catenin (Cat # 06-734) and FDPS (Cat # HPA028200), and rabbit monoclonal FGFR1-Ab1 (Cat # ZRB1243) that detects the N-terminal extracellular domain were from Sigma-Aldrich (St. Louis, MO, USA). Rabbit monoclonal FGFR1- Ab2 (Cat # ab76464) that detects the intracellular domain was from Abcam (Waltham, MA, USA). Mouse monoclonal antibodies against GM130 (Cat # 610823) and RalA (Cat # 610222) were from BD Biosciences (Franklin Lakes, NJ, USA). Mouse monoclonal anti-EGFR (Cat # MA5-13070) was from Thermo Fisher Scientific/Invitrogen. Mouse monoclonal anti-GFP (Cat # sc-9996) was from Santa Cruz Biotechnology (Dallas, TX, USA). Mouse monoclonal anti-β-tubulin (Cat # E7) and anti-stage specific embryonic antigen-1 (SSEA-1) (Cat # MC-480) were from Developmental Studies Hybridoma Bank (DHSB, Iowa City, IA, USA). Horseradish peroxidase (HRP)-conjugated goat anti-mouse and anti-rabbit secondary antibodies and donkey anti-mouse and anti-rabbit secondary antibodies were purchased from Sigma-Aldrich. Fluorescence-conjugated secondary antibodies including anti-mouse and anti-rabbit IgG antibodies raised in goat, donkey or chicken, conjugated to Alexa Fluor 488, Alexa Fluor 546 or Alexa Fluor 633, were purchased from Thermo Fisher Scientific/Molecular Probes. Cells were treated with pharmacological inhibitors, where indicated, 2 h prior to ligand stimulation. Inhibitor concentrations in DMSO (Cat # 276855, Sigma-Aldrich) were 40 μM for SU5402 (Cat # 572630, Sigma-Aldrich); 10 μM for AKT inhibitor VIII (Cat # 124018, Sigma-Aldrich); and 5 μM for U0216 (Cat # 662005, Sigma-Aldrich). DMSO alone was used as control at <1%. Surface biotinylation to detect cell surface R1-GFP in Nx, SS or Hx was performed as described by us previously [14]. Anti-GFP immunoaffinity capture was performed using Chromotek GFP-TRAP magnetic particles (Proteintech Group, Rosemont, IL, USA) according to manufacturer’s instructions with a modification of using post-20K fraction obtained from subcellular fractionation described above.

### 2.9. Plasmids and Recombinant DNA Constructs

RED is an optimized version of Anthomedusae jellyfish chromoprotein that does not aggregate. The coding sequence was subcloned from the parent vector pmaxFP-RED-N or pmaxFP-RED-C (Lonza). GFP used in this study is the EGFP (enhanced green fluorescent protein) encoded in the pEGFP vector (Clontech). The hFGFR1 IIIc cDNA was generated in our laboratory by RT-PCR from mRNA isolated from SK-RC-31 and cloned in pBluescript II SK(+) (Stratagene, San Diego, CA, USA). Full-length cDNA sequence was confirmed by DNA sequencing. Plasmids containing hFGFR2, mFGFR3, hFDPS and hRalA were purchased from Thermo Fisher Scientific/Open Biosystems. cDNA sequences of Rab2a, Rab4a, Rab5a, Rab6a, Rab7a, Rab8a, Rab9a, Rab11a, Rab13a, caveolin-1 and caveolin-2 were obtained in our lab by RT-PCR amplification from RNA isolated from non-cancerous human kidney HK-2 cells and cloned into pBluescript II SK(+) (Stratagene). Plasmids containing cDNAs corresponding to hRalA-G23V, hRalA-S28N, hGlut4, hRab8aQ67L, tubulin-GFP and EB1-GFP were from Addgene (Watertown, MA, USA). Kits containing Rab8a siRNAs (Cat # SR303866) and scrambled siRNAs (Cat # SR30004) were purchased from OriGene (Rockville, MD, USA). Oligonucleotides for PCR and RT-PCR were custom synthesized from Eurofins (Louisville, KY, USA). Recombinant constructs were cloned in pCDNA 3.1+ (Geneticin resistance; Cat # V79520, Thermo Fisher Scientific/Invitrogen) and pCDNA3.1 Hygro(+) (Hygromycin resistance; Cat # V87020, Thermo Fisher Scientific/Invitrogen) for co-expression. Full-length sequences of all engineered constructs expressing the recombinant proteins were confirmed by DNA sequencing.

### 2.10. mEB Immunofluorescence

mEBs were grown in suspension cultures as described previously [41]. At day 8 of suspension culture, mEBs were collected and settled by gravity in microcentrifuge tube, washed with ice-cold PBS, settled again by gravity and fixed in ice-cold 2% para-formaldehyde for 45 min on ice. After quenching with 10 mM glycine in PBS, mEBs were washed thrice with room temperature PBS. Subsequently, the mEBs were resuspended in agar to create a cell button and processed as a block for paraffin embedding. The paraffin-embedded mEB block was cut in 5 μm serial sections and mounted on glass slides. For immunofluorescence, mounted sections on glass slides were deparaffinized at 60 °C for 60 min followed by 10 min in xylene. The sections were then serially rehydrated (95–0% ethanol) and permeabilized with 0.25% saponin for 30 min. After incubation for 30 min in blocking solution (1% BSA + PBS), sections were incubated with primary antibody in blocking solution for 4 h. After three 5 min washes in PBS, sections were incubated with secondary antibody in blocking solution for 2 h. The sections were washed in PBS (3 × 5 min) and mounted in 25% glycerol.

### 2.11. Migration Assays

Pacman assay was carried out as follows: fibronectin-coated (2.5 μg/cm^2^) 60 mm tissue culture plates (see above) were covered with 1 μm carboxylate-modified polystyrene fluorescent microspheres (Cat # F8801, FLUOSPHERES, Thermo Fisher Scientific) at 0.005% in DPBS for 4 h at room temperature to allow coating of fluorescent microspheres. Cells subjected to SS or Hx for 24 h and 48 h were seeded at 4 cells/mm^2^ density in respective medium with added sterile 2% BSA. Seeded cells were incubated for an additional 16 h in SS or Hx in the presence of pathway-relevant pharmacological inhibitors from Sigma-Aldrich (SU5402, 40 μM and 60 μM; AKT inhibitor VIII, 10 μM; MEK1/2 inhibitor U0216, 5 μM; FGFR-VEGFR-PDGFR receptor tyrosine kinase inhibitor 3-(4-dimethylaminobenzylidenyl)-2-indolinone (DMBI), 40 μM and 60 μM). At the end of experimentation, cells were fixed in ice-cold 4% paraformaldehyde for 15 min and the migration tracks were imaged using Olympus FV300 with a 10× objective. The migrated tracks (cleared of fluorescent beads) were traced and measured using NIH ImageJ version 1.48.

Trans-well Boyden chamber (6.5 mm diameter, 8 μm pore size; Cat # CLS3422, Corning) assays were performed using 15,000 cells/well seeded into the upper chamber. For Hx, cells were maintained in 1.5% O_2_ for 24 h or 48 h prior to seeding into Boyden chamber. Similarly, SS cells were serum-starved for 24 h or 48 h and freshly seeded into Boyden chamber to initiate the assay. The migration towards serum-free medium containing 50 ng/mL hFGF2 (human basic FGF, Cat # SRP6159, Sigma-Aldrich) in lower chamber was followed for 16 h in either SS or Hx conditions. For inhibitor treatments, Boyden chamber inserts with seeded cells were treated with pharmacological inhibitors in DMEM with 1% FBS. Inserts were rinsed in PBS, fixed with 3.7% formaldehyde for 30 min and stained with 0.005% crystal violet for 1 h. Cells in the upper chamber were removed with a cotton swab and migrated cells attached to the underside of the membrane were counted using a 10× objective focused at the center.

### 2.12. Statistical Analysis

Experiments were repeated at least thrice. Independent Student’s *t*-test was used to compare continuous variables between two groups in Boyden chamber, Pacman and mEB beating assays. The alpha level of statistical significance was set at 0.05 for all tests. The average numbers of mEBs analyzed from 4 independent experiments for each condition are as follows: Nx = 45, Hx = 34, Nx + FGFR1 = 54, Hx + FGFR1 = 58, Nx + RalA = 37, Hx + RalA = 54, Hx + SU5402 = 42, Hx + U0126 = 50, Hx + AktVIII = 34, Nx + SU5402 = 38, Nx + U0126 = 40, Nx + AktVIII = 35, Nx + FGFR1 + C1 = 42, Nx + FGFR1 + C3 = 31, HX + C1 = 37, HX + C3 = 31, NX + FDPS-RED = 23, NX + RED-FDPS = 32, HX + FDPS-RED = 35 and HX + RED-FDPS = 38. Results for mEB experiments are expressed as percentage of beating mEBs over total mEBs.

## 3. Results

### 3.1. Characterization of Intracellular FGFR1

**Figure 1:**
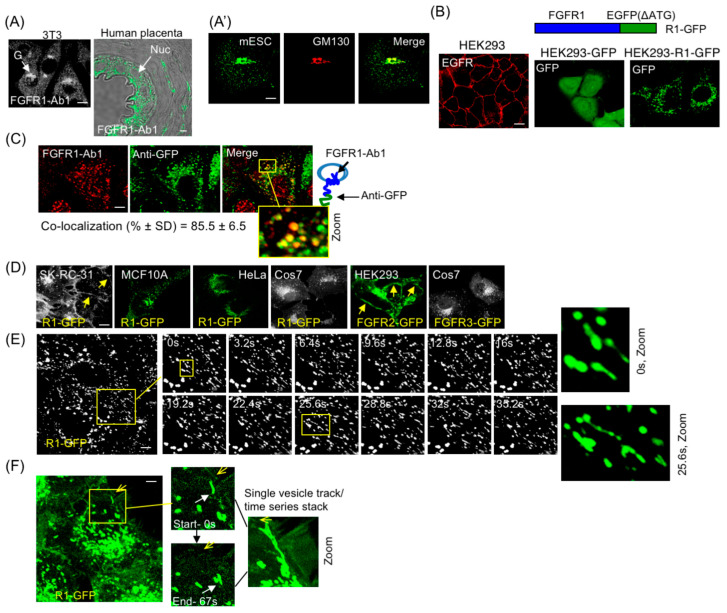
FGFR1 localization and identification of RART vesicles. (**A**) Endogenous FGFR1 is localized to intracellular vesicles in NIH3T3 (3T3) and human placenta (**A**), and mESC (**A′**), as detected with FGFR1-Ab1 antibody (detecting extracellular or vesicle luminal domains) under indirect immunofluorescence (IF). The mESC samples were co-stained with FGFR1-Ab1 and *cis*-Golgi marker GM130. G, Golgi; Nuc, nucleus. (**B**) IF shows EGFR on the PM in HEK293 cells (left panel). FGFR1-EGFP fusion protein (R1-GFP) is depicted (top right). Staining for GFP for stable expression of GFP in HEK293-GFP cells (middle panel) or HEK293-R1-GFP cells (right panel) shows cytosolic and vesicle localization, respectively. (**C**) Stable HEK293 cells expressing R1-GFP were fixed and co-stained with FGFR1-Ab1 and anti-GFP. FGFR1- and GFP-positive vesicles mostly overlap in punctates. (**D**) Transient expression in indicated cells showing intracellular vesicle pattern of R1-GFP in *VHL^+^* epithelial cells. R1-GFP in SKRC (*VHL^−^*) and FGFR2-GFP in HEK293 are PM localized and served as positive controls (yellow arrows). R1-GFP in MCF10A and HeLa, and FGFR3-GFP in Cos7 are localized in vesicles. (**E**) Single-plane time-lapse confocal imaging shows dynamic R1-GFP vesicles extending tubules. The two zoomed images on the right are from boxed regions in 0 s and 25 s. (**F**) Single vesicle tracking (white arrows in enlarged views of boxed area point to the tips of the migrating vesicles) shows a vesicle approaching the PM and subsequently being propelled back to cell interior. Sharp yellow arrows point to the PM location closest to the vesicle of interest. The entire stack of vesicle tracks is shown in zoomed image on the right. Five independent experiments were performed and one representative example is shown for each assay. Yellow boxes are regions of interest that are enlarged (Zoom). Bars are 20 μm.

Like all RTKs, FGFR1 IIIc contains a single transmembrane domain with the N-terminus facing vesicle lumen (or extracellular space) and C-terminus exposed to cytosol. For simplicity, FGFR1 IIIc is mentioned as FGFR1 in this study. In FGFR1-EGFP fusion (R1-GFP), the initiating ATG codon of EGFP open reading frame was deleted to prevent internal translation initiation. NIH 3T3 cells, polarized epithelium of human placental tissue and mESCs were used to examine endogenous FGFR1 intracellular localization. FGFR1 in NIH3T3 showed a Golgi and vesicle localization pattern and human placenta tissue showed mainly vesicle location, as detected by N-terminus-specific FGFR1-Ab1 antibody (Figure 1A). In the case of mESCs, FGFR1 expression in mESCs also showed an intracellular vesicle pattern in addition to localization in Golgi, as shown by co-localization with the *cis*-Golgi marker GM130 (Figure 1A′). Golgi localization of FGFR1 in NIH 3T3 or mESCs may be related to their mesenchymal or undifferentiated state, since efficient PM localization requires close cell–cell contact. Therefore, in our subsequent study, cells were typically allowed to grow in clusters to observe PM localization, as confirmed by proper PM localization of epithelial growth factor receptor (EGFR) in HEK293 cells (Figure 1B, left panel).

Several previous studies utilized fluorescent proteins fused to C-terminus of FGFR1 to study signaling and receptor endocytosis. We also employed R1-GFP fusion to study FGFR1 subcellular localization (Figure 1B, top). In epithelial HEK293 stable cell line expressing GFP alone or R1-GFP fusion (Figure 1B, middle and right panels, respectively), GFP alone showed diffused cytosolic expression while R1-GFP showed punctate staining typical of vesicle localization. Co-staining of R1-GFP expressing HEK293 cells with anti-GFP antibodies and FGFR1-Ab1 confirmed that the vesicle-associated GFP fluorescence corresponded to FGFR1 (Figure 1C). SK-RC-31 is a *von Hippel-Lindau* (*VHL*) mutant cancer cell line that excessively accumulates FGFR1 on PM as a result of defective endocytic internalization of surface FGFR1 [14]. The observed PM localization of R1-GFP in SK-RC-31 cells confirmed that the receptor fusion was indeed capable of cell surface localization (Figure 1E, left panel). R1-GFP was present in intracellular vesicles in multiple other *VHL*^+^ cell lines examined (MCF10A, HeLa, Cos7) (Figure 1D). FGFR2-GFP was PM-localized, while FGFR3-GFP was in vesicles in HEK293 and Cos7 cells, respectively (Figure 1E, right two panels) as previously reported [42], indicating receptor subtype-specific regulation of PM localization of different FGFRs.

In the following sections, R1-GFP-containing vesicles will be referred to as the R1 vesicles. Time-lapse microscopy showed the dynamic nature of the R1 vesicles. The R1 vesicles projected tubular extensions and exhibited curvilinear motion (Figure 1E; Supplementary Video S1). Single-vesicle tracking detected the R1 vesicles approaching the PM and moving back to the cell interior without undergoing PM fusion (Figure 1F; Figure S1; Supplementary Video S2). This indicates that in an uninduced state, FGFR1-containing vesicles cannot deliver the receptor to the PM, although the vesicles can be transported to the juxta-membrane position.

### 3.2. Identification of R1 Vesicles as a Novel Compartment

**Figure 2:**
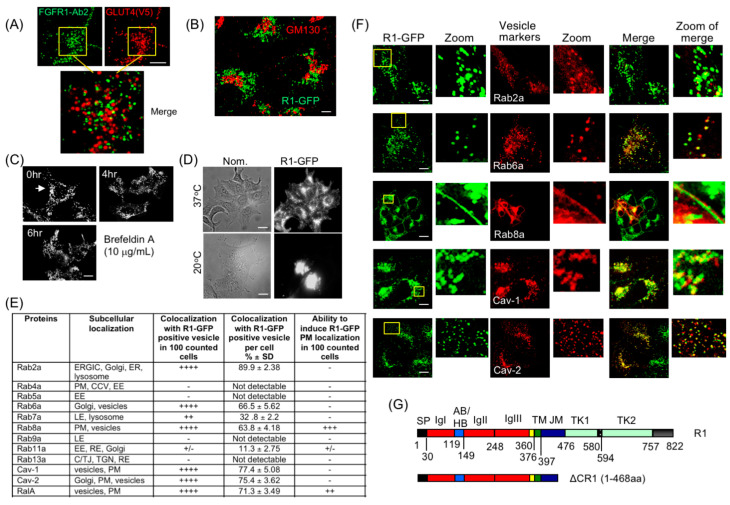
Characterization of the RART vesicles. (**A**) R1-GFP-containing vesicles do not overlap with GLUT4-positive vesicles. HEK293-R1 cells co-expressing R1-GFP and V5 epitope-tagged GLUT4 were fixed. R1-GFP was stained with FGFR1-Ab2 (detecting intracellular domain) and GLUT4 detected with anti-V5 antibody. The two molecules do not overlap. The two boxed areas are enlarged and merged in the lower panel. (**B**) HEK293-R1 cells were co-stained with anti-GFP (green) and anti-GM130 (marking Golgi; red). The R1 vesicles do not overlap significantly with GM130-positive staining. (**C**) Stability of R1-GFP vesicles was confirmed by Brefeldin-A treatment, which resulted in collapse of Golgi (arrow) but failed to disrupt R1-GFP localization pattern. (**D**) Transit through ER-Golgi was confirmed by 20 °C low-temperature Golgi-block in HEK293-R1 cells, which accumulated R1-GFP in Golgi at 20 °C (bottom right), compared to 37 °C (top right). Bright-field (with Nomarski optics) views of the same cell populations are shown on the left (Nom.). (**E**) Subcellular localizations of RED fusions of Rabs, caveolins and RalA expressed in HEK293-R1-GFP are summarized, based on representative results shown in (**F**) and in Supplementary Figure S2. Co-localization of R1-GFP with Rab2a, Rab6a, Rab8a, RalA and caveolins is observed. Other proteins tested did not co-localize with the R1 vesicles nor alter R1-GFP localization (Figure S2A). Co-localization and ability to induce R1-GFP PM localization was assessed from live-cell time-lapse imaging immediately followed by re-imaging cells after brief fixation (5 min, PBS + 2% paraformaldehyde). Table in (**E**) presents summary results of RED fusions’ co-localization with R1-GFP (column 3) and their ability to induce FGFR1 PM localization (column 4) when co-expressed with R1-GFP. CCV, clathrin-coated vesicle; A/TJ, adherens/tight junction; EE, early endosome; ERGIC, ER-Golgi intermediate compartment; LE, late endosome; RE, recycling endosome; TGN, trans-Golgi network. Quantification was performed as described in “Quantification of microscopic images” of “Materials and Methods”. (**F**) Representative results of RED fusions of Rabs and caveolins expressed in HEK293-R1. The R1 vesicle localization patterns are summarized in (**E**). Boxed areas in the R1-GFP panels are enlarged as the “Zoom” insets. The same zoomed areas in “Vesical markers” and “Merge” are also shown. (**G**) Schematics and domain structure of full-length FGFR1 and the C-terminal deletion of ΔCR1. SP: signal peptide; IgI, IgII, IgIII: IgG-like domains; AB: acidic box; HB: heparin-binding domain; TM: transmembrane domain; JM: juxtamembrane domain; TK1, TK2: split tyrosine kinase domains. Representative images from five independent experiments are shown. Bars are 20 μm.

One known example of vesicle-localized surface protein is GLUT4, a glucose transporter. It is sequestered in cytoplasmic vesicles and present at low levels on the PM (~5%) in steady state [43]. Insulin stimulation induces GLUT4 translocation to the PM by vesicle exocytosis. This process is rapid and results in 50% of the receptor being translocated to the PM within 30 min after insulin stimulation. To examine whether the R1 vesicles utilize the same vesicle transport mechanism, we co-expressed V5 epitope-tagged GLUT4 in HEK293 cells stably expressing R1-GFP (HEK293-R1 cells) and observed that R1 vesicles were distinct from GLUT4-positive vesicle population (Figure 2A). A 30 min stimulation of HEK293-R1 cells with FGF2, a high-affinity ligand of FGFR1, or with insulin or histamine, failed to induce detectable PM translocation of R1-GFP, indicating that these external stimuli do not alter the R1 vesicle dynamics to initiate PM localization. Next, we addressed the origin of the R1 vesicles. Co-staining with the *cis*-Golgi marker, GM130, showed that the majority of the R1 vesicles were localized to the post-Golgi compartment in the HEK293 cells (Figure 2B). Consistent with this, the existing pattern of the cytosolic R1 vesicles remained unaltered upon treatment with Brefeldin-A, a Golgi-disrupting agent (Figure 2C). This indicated that the R1 vesicles represented a steady-state post-Golgi pool. When HEK293-R1 cells were incubated at 20 °C for 3.5 h in a typical Golgi-block assay (i.e., blocking anterograde Golgi-to-cytosol transit at low temperature), R1-GFP accumulation within the Golgi is greatly increased with concomitant decrease in the vesicle pool (Figure 2D), additionally indicating that the R1 vesicles originate from Golgi, and that they are stable for at least 6 h after Golgi is disrupted (Figure 2C).

To further obtain clues to their identity and fate, cellular markers for the R1 vesicles were sought. An excellent body of previously published work has shown that small GTPases control formation and delivery of vesicles [44]. In particular, different Rab GTPases are recruited to specific sub-populations of vesicles and are widely used as markers to distinguish between different vesicle compartments [45,46]. Using indirect immunofluorescence (IF) in HEK293-R1 cells, we tested the presence of various endogenous Rab proteins and other known vesicle markers (listed in Figure 2E). Selected results are presented (Figure 2F and Figure S2A,B) and summarized in Figure 2E. The outcome of these experiments revealed that the R1 vesicles contain Rab2a, Rab6a, Rab8a and caveolins (Figure 2F), but not Rab4a, Rab9a or Rab13a (Figure S2A). There is a very small fraction of the R1 vesicles co-expressing Rab5a (Figure S2A), likely representing clathrin-coated vesicles or early endosomes [14,23]. Interestingly, RalA co-localizes with FGFR1, and can be seen to co-localize with FGFR1 on the PM when RalA is overexpressed, while RalB only partially co-localizes with FGFR1 and does not localize to the PM (Figure S2B, also see below). Although these two Ral isoforms are structurally similar, RalA is more involved in anchorage-independent cell growth, vesicle trafficking and cytoskeletal organization [47,48]. We therefore focused on RalA in this report.

Thus, collective presence of Rab2a, Rab6a, Rab8a, RalA and caveolin markers constitute a hitherto unreported conglomeration of such factors and, therefore, represent a novel FGFR1-containing PGV population.

### 3.3. Intracellular FGFR1 Vesicles Are Stably Maintained

The FGFR1 C-terminal scaffold is known to interact with various signaling molecules. We therefore generated a C-terminal deletion of FGFR1 to test if it played a role in vesicle localization (Figure 2G). In ΔCR1-GFP (1–468 aa), only a short 69 aa stretch of cytosol-exposed FGFR1 juxtamembrane domain is retained, removing aa residues 469–822 from FGFR1 C-terminus that completely encompasses the split tyrosine kinase domains. Similar to R1-GFP, the ATG-initiating codon of EGFP in ΔCR1-GFP was removed. R1-GFP or ΔCR1-GFP was then exogenously expressed in HEK293 cells. HEK293 cells expressing R1-GFP and ΔCR1-GFP will be referred to as HEK293-R1 and HEK293-ΔCR1, respectively. In HEK293-R1 and HEK293-ΔCR1 cells, cycloheximide (CHX) treatment for various durations to block protein translation showed approximately 45% of R1-GFP degraded by 4 h as assessed by Western blotting (Figure S3A) and quantified in Figure S3A′, presumably via lysosomes since a portion of the R1 vesicles co-localized with Lysotracker (Figure S3A″), while a 55% fraction of R1-GFP persisted up to 8 h post-CHX treatment (Figure S3A,A′). By contrast, ΔCR1-GFP was fully degraded within this time frame (Figure S3A,A′), suggesting that FGFR-1 C-terminus conferred stability, and influenced receptor steady state levels. At the CHX concentration used, protein synthesis block occurs within 3–4 h in HEK293 cells [49]. Furthermore, at 6–8 h CHX chase time, when a stable fraction existed (Figure S3A), R1-GFP was present in vesicles (Figure S3A‴).

These results suggest that the R1 vesicles (1) can be identified with protein markers known for their properties to localize to vesicle sub-populations, and (2) contain an FGFR1 receptor that exists without requiring ongoing protein synthesis, representing a stably sequestered pool. This conclusion is consistent with the result showing the stable post-Golgi pool of the R1 vesicles (Figure 2C).

### 3.4. PM Delivery of FGFR1 Is Induced by Starvation and Hypoxia

It is known that FGFR1 activation occurs at the PM after binding to extracellular ligands. If cellular FGFR1 is sequestered in intracellular vesicles, what is its physiological significance and how is the receptor delivered to the PM? To address this, we turned to the R1 vesicle components for answers. We hypothesized that exogenous expression of the above-identified R1 vesicle components might promote or inhibit PM translocation of FGFR1 and potentially point to the regulatory mechanism. The results of such experiments are discussed below. RalA showed co-localization with R1-GFP at the PM, and exogenous expression of RalA further induced PM localization of R1-GFP (Figure S2B, top panels; Figure 2E). Besides RalA and among the Rab proteins that localize to the R1 vesicles, only the exogenous expression of Rab8a showed a strong induction of PM localization of R1-GFP. Exogenous expression of other Rab proteins tested did not alter R1-GFP localization in HEK293-R1 cells (summarized in Figure 2E), suggesting that Rab8a and RalA may play specific roles in FGFR1 PM translocation. It is known that Rab8a is involved in apical and basolateral sorting and in nutritional response [50]. In addition, FGFR’s well-known role in branching morphogenesis is intimately linked to hypoxia response [51,52]. Therefore, we next tested if serum starvation (SS, no FBS) or hypoxia (Hx, 1.5% O_2_) could activate PM translocation, thus possibly pointing to the biological relevance.

**Figure 3:**
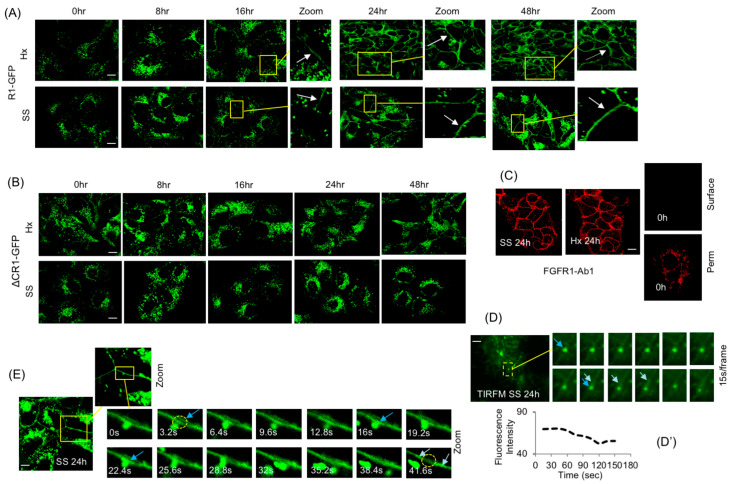
PM translocation of FGFR1. (**A**) Incubation of HEK293-R1 cells in serum starvation (SS) or hypoxia (Hx) for indicated durations showing progressive PM localization of R1-GFP (arrows). (**B**) SS or Hx did not induce PM localization of ΔCR1-GFP within the 48 h observation period. (**C**) Indirect IF with FGFR1-Ab1 was used to confirm surface localization of the receptor in HEK293-R1 cells in 24 h SS (left panel) or Hx (middle panel) under non-permeabilizing conditions (SS 24 h and Hx 24 h). Right: same cells at 0 h were examined similarly and no cell surface localization of R1-GFP was detectable (upper right panel; 0 h, surface), but when permeabilized (lower right panel; 0h, perm), R1-GFP was detectable in intracellular vesicles. (**D**) TIRF microscopy (TIRFM) showing the fusion of an R1 vesicle in 24 h SS (SS 24 h, dashed circle). The fusion event is shown in time series of frames taken at 15 s intervals (from top left to bottom right; blue arrows). Double arrow shows the point of fusion on the PM when vesicle loses fluorescence (quantification in **D′**) and the PM gains diffused fluorescence (light blue arrow), indicating transfer of receptor from vesicle to the PM. (**D′**) The fluorescence intensity measurements of (**D**) using ImageJ revealed typical fusion event where partial content of R1 vesicle is transferred to the PM (see text for details). The boxed area is shown in the “Zoom” images of time series (left-to right and up to down). (**E**) Time-lapse imaging at single confocal plane in 24 h SS (SS 24 h) shows that a vesicle approaches and contacts the PM via extending tubule and delivers the receptor (increase in fluorescence at the delivery spot indicated by blue arrow at 3.2 s, 16 s and 22.4 s). After delivery, the receptor is seen spreading laterally on the PM (41.6 s, indicated by light blue arrows). The circled portion initially shows PM delivery (3.2 s) and in 41.6 s shows loss of fluorescence at delivery spot due to lateral spreading of receptor. The boxed area is shown in the “Zoom” images of time series. Representative images from five independent experiments are shown. Bars are 20 μm.

Standard culture condition is referred to as normoxia (Nx). Exposure of HEK293-R1 cells to SS or Hx caused a significant shift in R1-GFP localization to the PM; R1-GFP cell surface levels were detectable starting at 16 h in both Hx and SS conditions, and increased up to 48 h without significant increase in R1-GFP total protein level (Figure 3A). By contrast, PM localization of ΔCR1-GFP was undetectable in SS or Hx under identical experimental conditions described above (Figure 3B). Therefore, the C-terminus of FGFR1 mediates PM localization in response to environmental changes such as hypoxia and starvation.

The PM presence of R1-GFP in SS or Hx was confirmed using FGFR1-Ab1, an N-terminus-directed antibody, under non-permeabilizing conditions after quenching R1-GFP fluorescence with paraformaldehyde (Figure 3C, top panels). Cell surface presence of R1-GFP at 0 h was undetectable under the same non-permeabilizing conditions (Figure 3C, upper right panel); however, under permeabilizing conditions (Figure 3C, lower right panel), after quenching GFP fluorescence, an intracellular vesicle pattern was seen at 0 h, as expected, again confirming the presence of an intracellular pool of FGFR1. TIRFM, employed to observe vesicle dynamics at the PM, detected R1 vesicle fusions with the PM at 24 h SS (Figure 3D). Fluorescence measurements of TIRFM images (Figure 3D′) revealed typical phases of vesicle contact and cargo transfer described before for PM fusion and for cargo-sorting events [53,54]: phase I, R1 vesicle contacted the PM for 45–60 s; phase II, R1 vesicle lost 25% fluorescence over the next 50 s, with corresponding transfer of fluorescence to the PM and its lateral spread on the PM; phase III, after an additional 40 s at the PM, the R1 vesicle moved away. Single-plane confocal imaging also detected R1 vesicles undergoing PM fusions that mirrored the above-discussed ‘kiss-and-run’ transfer (Figure 3E; Supplementary Video S3).

### 3.5. The Sequestered FGFR1 Pool Is Functional in PM Translocation

**Figure 4:**
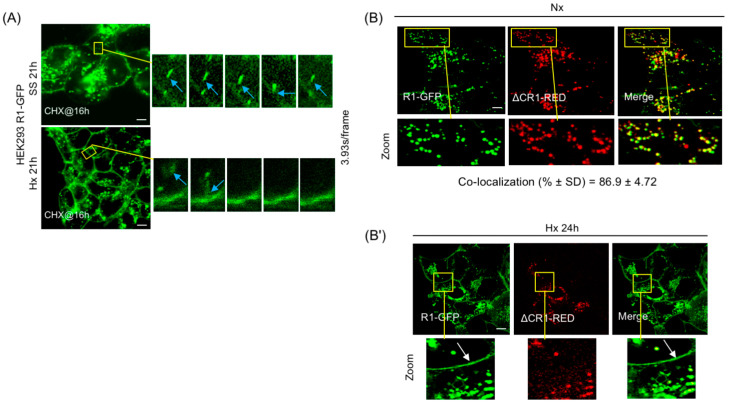
Influence of the RART pathway in FGFR1-mediated signaling in SS and Hx. (**A**) Kiss-and-run fusion of R1-GFP with the PM is observed at 21 h of SS (top, blue arrow) and Hx (bottom, blue arrow) that included 5 h of CHX treatment, showing that the stable R1 vesicle pool is the source of receptor delivery to the PM. CHX was added at 16 h of SS and Hx. The boxed areas are enlarged on the right and shown in time series. (**B**) R1-GFP (green) and ΔCR1-RED (red) are co-localized in the R1 vesicles in HEK293 cells, showing that ΔCR1 contains the domains necessary to properly localize to the R1 vesicle. In 24 h Hx (**B′**), only R1-GFP (green, arrow) is localized on the PM, indicating that PM delivery requires C-terminus of FGFR1 and does not involve full vesicle fusion to the PM. Representative data from five independent experiments are shown. Yellow boxes are regions of interest that are enlarged (Zoom). Bars are 20 μm.

In order to verify whether FGFR1 already present in the R1 vesicle pool is utilized or if freshly synthesized FGFR1 is required for PM delivery, HEK293-R1 cells were first subjected to 16 h of SS or Hx. At 16 h of SS or Hx, the R1 vesicles presumably gained competency for PM fusion as judged by low but detectable PM levels of R1-GFP at this time point (Figure 3A). CHX experiments independently showed that, after 4 h treatment, the existing R1 vesicle pool consisted predominantly of post-translational sequestered R1-GFP vesicle fraction (see above, Figure S3A‴). Therefore, we performed a combined treatment by first subjecting HEK293-R1 cells to 16 h of SS or Hx to generate fusion competent R1 vesicles, followed by 5 h of CHX treatment to block new synthesis of the receptor. Time-lapse confocal imaging of cells at 21 h of this treatment (16 h SS/Hx + 5 h CHX chase under continued SS or Hx) detected R1 vesicle fusions at the PM and increased cell surface levels of R1-GFP in SS and Hx (Figure 4A; Supplementary Video S4 and Supplementary Video S5), suggesting that the FGFR1 sequestered in vesicles represented a functional pool for PM delivery.

### 3.6. PM Translocation Requires FGFR1 C-Terminus and Does Not Involve Full Vesicle Fusion

The lack of PM localization of ΔCR1-GFP in SS and Hx might be explained if ΔCR1 was mis-targeted to vesicles distinct from the R1 vesicles or if ΔCR1 truly lacked the domains required for PM translocation. To verify this, R1-GFP and ΔCR1-RED were transiently co-expressed in HEK293 cells (Figure 4B). ΔCR1-RED and R1-GFP co-localized almost completely in PGV with each other, suggesting that deletion of aa 468–822 cytosolic domain of FGFR1 did not affect correct targeting of ΔCR1 to the R1 vesicle. Using these recombinant proteins, we tested if full fusion of the vesicle was the operating mechanism for PM delivery. If delivery by full vesicle fusion occurred, cells co-expressing ΔCR1-RED and R1-GFP should display both ΔCR1-RED and R1-GFP on the PM when shifted to Hx for 24 h. However, if receptor partitioning occurs within the R1 vesicle subdomain en route to the PM for delivery, we expect R1-GFP alone to be present on the cell surface. Remarkably, R1-GFP localization was detected on the PM by 24 h of Hx, but ΔCR1-RED remained localized to the R1 vesicles and not present on the PM (Figure 4B′). The result revealed three aspects of the PM translocation mechanism of FGFR1: (1) the R1 vesicles are the source for PM localization of FGFR1, (2) FGFR1 C-terminus is critical for PM localization, not vesicle targeting, since ΔCR1 remained in the R1 vesicles, and (3) most importantly, the mechanism does not involve full fusion of the R1 vesicle to the PM since only R1 translocated to the PM while co-localized ΔCR1 remained in the vesicles. Our results above indicate that mechanistically, FGFR1 is partitioned into distinct sub-vesicular domains where localized assembly of RART pathway components occurs to facilitate kiss-and-run delivery of the receptor to the PM.

### 3.7. FGFR1 on the PM Is Functional and Influences Cell Behavior

Two assays were employed to quantify cell motility (Figure S4): a Pacman assay can trace the paths of migrating cells. However, since addition of FGF2 or serum led to non-directional migration, this assay was not useful for quantification of chemotactic cell movement (Figure S4A). For the latter purpose, a Boyden chamber assay was employed. A Pacman assay detected four- and sixfold increases in cell motility in 24 h and 48 h of SS, respectively, with R1-GFP, but importantly, not with ΔCR1 (Figure S4B). Relevant pharmacological inhibitors were used, including SU5402 against the FGFR1 activity, U0126 against the MEK1/2 activity, DMBI against the receptor tyrosine kinase activity and Akt VIII against the Akt activity. The SS-stimulated cell migration in the presence of R1-GFP was inhibited by DMBI, SU5402, U0126 and AKT VIII (Figure S4B,B′). Inhibition with DMBI and SU5402 confirmed FGFR1-dependent migration. In Boyden chamber assays, FGF2 stimulation led to two- and threefold increase in 24 h and 48 h of SS, respectively, which was inhibited by SU5402, U0126 and AKT VIII (Figure S4C). In addition, in Boyden chamber assay, cell invasion stimulated by FGF2 treatment was further increased by Hx for 24 h and 48 h, and SU5402, U0126 and AKT VIII inhibited this invasive behavior (Figure S4D).

A similar pattern was obtained in SS and Hx; therefore, only representative results are discussed in the following sections to avoid repetitive presentation of voluminous data. Experiments were performed at least three or four independent times, with similar results, to support the conclusions discussed below, and only representative results are shown in figures. Direct measurements of FGFR activation, using phospho-FGFR1 antibodies to detect activated FGFR (in the form of R1-GFP, Figure S4E), showed increase in FGF2-stimulated receptor activity by ~2.2-fold, relative to total R1-GFP levels, at 48 h of Hx, compared to pre-treatment (0 h, quantified in Figure S4E′). FGF2 stimulation increased Erk1/2 activity by 2.5-fold in Hx compared to Nx and is reversed by SU5402 (compare 48 h with 0 h in Plus FGF2 panel, Figure S4F). Note that there is a general decline in total FGFR protein level but the portion of phospho-FGFR over total protein continued to rise. Interestingly, SU5402 did not significantly affect the p-AKT levels with or without FGF2 stimulation in Hx (Figure S4F), suggesting that in our culture conditions in HEK293 cells, AKT activation is not significantly upregulated by FGFR signaling. However, given AKT’s role in cell motility, results shown in our assays above (Figure S4B–D) could indicate that the basal levels of AKT function are sufficient for FGFR1-mediated cell motility. These combined results indicate that FGFR1-mediated signaling and cell migration is directly linked to increased PM localization in SS and Hx.

### 3.8. FDPS Binds FGFR1 C-Terminus and Is Involved in the PM Translocation Mechanism

**Figure 5:**
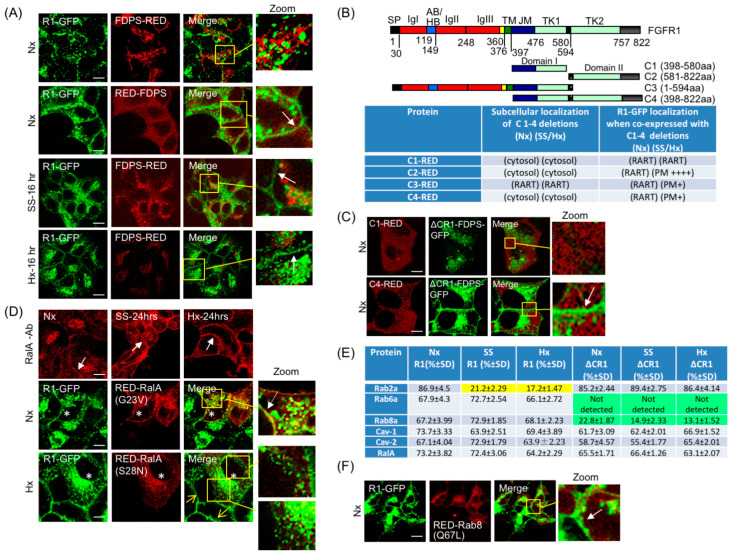
Regulators of FGFR1 PM localization require the receptor C-terminus. (**A**) Co-expression of R1-GFP (green) and FDPS-RED (red) in HEK293-R1 cells shows FDPS presence in ER in Nx and is largely non-overlapping with R1-GFP (top row). RED-FDPS fusion was engineered to block N-terminus ER localization motif and force cytosolic localization of FDPS. RED-FDPS expression increased PM localization of R1-GFP in Nx in HEK293-R1 cells (second row, arrow). FDPS-RED presence in cytosol is increased in 16 h SS or Hx, at which time PM translocation of R1-GFP is detectable (third and fourth rows, respectively, arrows). (**B**) Expression of indicated FGFR1 deletions (schematics on top, names in column 1) and their influence on PM localization of R1-GFP in Nx, Hx and SS are summarized in Table. Following transient expression in HEK293 parental cells, subcellular localization of FGFR1 deletions, C1-4, was studied in Nx, SS and Hx, and summary of these results are shown in column 2. Next, the deletion constructs were expressed in HEK293-R1 cells and their effects on R1-GFP localization in Nx, SS and Hx were studied, and results are shown in column 3 (RART = R1 vesicle). See Figure 2G legend for domain designation. (**C**) FDPS localization to the R1 vesicle using ΔCR1-FDPS-GFP chimera results in C1-RED recruitment to the RART vesicles (top row), although no detectable PM localization of ΔCR1-FDPS-GFP was seen in Nx. C4-RED expression, which contains full FGFR1 C-terminus, induced PM localization of ΔCR1-FDPS-GFP (bottom row, arrow). (**D**) After fixation and indirect IF with RalA antibody, increased PM association of endogenous RalA was observed in SS and Hx (top row; compare middle and right panels with left panel, arrows) in HEK293-R1 cells. In cells transiently expressing constitutively active RalA-G23V (asterisks), PM localization of R1-GFP is detected in Nx (second row, arrow in inset), compared to neighboring cells that do not express RalA-G23V and show no PM localization in Nx. Conversely, cells expressing dominant-negative RalA-S28N (asterisks, third row) show inhibition of R1-GFP PM localization in Hx (third row, insets), while neighboring non-expressing cells show PM localization (sharp yellow arrows). (**E**) HEK293-R1 and HEK293-ΔCR1 cells were transfected with indicated RED fusions (column 1). Localization of Rab2a, Rab6a, Rab8a, Caveolin-1, Caveolin-2 and RalA was examined in Nx, SS and Hx and the influence of FGFR1 C-terminus was assessed. The Table summarizes semi-quantitative observations of presence (+) or absence (−) of these proteins in the R1 or the ΔCR1 vesicles. Strongest presence is indicated by ++++. Color-shaded values indicate low or no localization (yellow for the R1 data and green for the ΔR1 data). (**F**) Expression of constitutively active RED-Rab8a(Q67L) in HEK293-R1 cells stimulates PM localization of R1-GFP in Nx (arrow). Quantification in (**B**) was performed as described in Section 2.7. Quantitation in (**E**) was performed as described in Section 2.12. Yellow boxes are regions of interest that are enlarged (Zoom). Bars are 20 μm.

In the yeast two-hybrid interaction assay, we identified farnesyl diphosphate synthase (FDPS/FPPS) as an FGFR1 interacting partner. A previous study showed increased physical association between FDPS and FGFR1 in starvation [55], but the precise cellular location of the association and its implications to FGFR1 activity remained unknown. FDPS enzymatic activity provides substrates for geranylgeranylation, a lipid modification required by Rab proteins for membrane association; therefore, a direct or indirect role of FDPS in the RART pathway is plausible. In order to test the relevance, we expressed N- and C-terminal fusions of FDPS with RED fluorescent protein to examine their effect on the RART pathway dynamics. FDPS contains the putative XXRR-like ER retention motif PLSR at the N-terminus, which remains functional when the RED fluorescent protein is fused at the C-terminus (FDPS-RED). When FDPS-RED is expressed in HEK293 and the ER-retention motif is exposed at the N-terminus, the fusion protein is found predominantly ER-localized in Nx, as expected (Figure 5A, top row). Furthermore, FDPS-RED expression did not co-localize with R1-GFP and did not alter R1-GFP localization in Nx. Thus, when FDPS is ER-localized, its expression level has little effect on the RART dynamics. However, when the RED fluorescent protein is fused at the N-terminus (RED-FDPS) where the ER retention motif of FDPS was masked, expression of the RED-FDPS chimera resulted in cytosolic localization and led to increased R1-GFP presence on the PM in Nx (Figure 5A, second row). Furthermore, at 16 h of SS or Hx, FDPS-RED exhibited a clear shift in subcellular localization from ER to cytosol in a punctate pattern in HEK293-R1 cells concomitant with the PM localization of R1-GFP, indicating that induced cytosolic localization of FDPS may be a prominent step in promoting FGFR1 PM translocation (Figure 5A, third and fourth rows).

To detect FGFR1 binding with endogenous FDPS, vesicles containing R1-GFP or ΔCR1-GFP were isolated from respective cells subjected to Nx or SS for 16 h. Anti-GFP immunoaffinity magnetic beads were employed to capture GFP-positive vesicles from post-20k differential centrifugation fraction and the association of endogenous FDPS with the isolated vesicles was detected by Western blotting. Under the isolation and wash conditions employed, anti-GFP immunoaffinity beads captured receptor-GFP fusions from R1-GFP or ΔCR1-GFP cells in Nx, as confirmed by FGFR1 Ab3 antibody detection on Western blots (Figure S5A, lanes 1 and 2). Using HEK293 cells expressing only GFP in SS or Hx, we did not detect any non-specific bands with FGFR1 Ab3 antibody on Western blots, confirming the specificity of the anti-GFP immunoaffinity capture method used (Figure S5A, lanes 3 and 4). The affinity capture assay detected FDPS association with the R1 vesicles under SS but not under Nx (Figure S5A′, compare lane 3 with lane 1). In addition, FDPS was not associated with the ΔCR1 vesicles under identical SS conditions (Figure S5A′, lane 2). Therefore, FDPS is associated with FGFR1 C-terminal region spanning aa 469–822, and the association occurs in an induced state such as SS. This is consistent with the result in Figure 5A.

To confirm in another way, we sought to dominantly interfere with FDPS binding to R1-GFP by expressing different FGFR1 deletions that span different portions of the C-terminal domain (Figure 5B): C1-RED contains aa 398–580; C2-RED contains aa 581–822; C3-RED contains the full N-terminal extracellular domain and the C-terminal domain up to aa 594; and C4-RED contains aa 398–822. We predicted that in SS and Hx, one or more of these fusion proteins, either in cytosol or on membrane, would soak up endogenous FDPS, and thus dominantly interfered with R1-GFP PM localization. Interestingly, expression of C1-RED could strongly inhibit R1-GFP PM localization in HEK293-R1 cells in SS and Hx; C3-RED and C4-RED modestly inhibited; while C2-RED could not (summarized in Figure 5B, Table). Thus, the results provide evidence that FDPS binding to FGFR1 is relevant for PM translocation. Considering the above observations together, we also infer that the FDPS binding domain in FGFR1 is between aa 469 and aa 580; i.e., ΔCR1 (1–468) does not bind FDPS (Figure 3B and Figure S5A) and C1 peptide (398–580) can soak up FDPS (Figure 5B).

To specifically test the function of FDPS, we designed a method to localize FDPS to the R1 vesicle as ΔCR1 fusion, using the ΔCR1-FDPS-GFP chimera. This is based on the finding that ΔCR1 can target to the FGFR1-containing (RART) vesicles without being translocated to the PM (Figure 4B). ΔCR1-FDPS-GFP was then co-expressed with C1-RED peptide (containing FGFR1 aa 398–580) or C4-RED peptide (containing FGFR1 aa 398–822), both containing the FDPS-binding domain (Figure 5B, top panel). We tested if C1 or C4 binding to ΔCR1-FDPS-GFP on RART vesicle is functionally equivalent to providing FGFR1 C-terminus in-trans, and if it would lead to PM localization of ΔCR1-FDPS-GFP. Our data showed that ΔCR1-FDPS-GFP failed to localize to the PM in Nx when co-expressed with C1-RED (Figure 5C, upper row). On the other hand, C4-RED, containing complete FGFR1 C-terminus of 398–822 aa, was recruited to the R1 vesicle and strongly induced PM localization of ΔCR1-FDPS-GFP in Nx (Figure 5C, lower row), providing the evidence that the full C-terminus of FGFR1 functions as a scaffold for FDPS binding and assembly of other factor(s) necessary for PM delivery. The C4-RED effect on ΔCR1-FDPS-GFP is unlikely the result of kinase activity because FGFR1 kinase inhibitor SU5402 did not inhibit PM translocation of R1-GFP in Hx or SS at 24 h (Figure S5B). In summary, (a) the aa 469–580 domain (domain-I) of FGFR1 binds FDPS, and (b) the aa 581–822 domain of FGFR1 C-terminus, referred to as domain-II here, is also required for PM translocation.

### 3.9. RART Pathway Components Function as Molecular Switches

Next, we sought to identify protein factor(s) that cooperate with FDPS in the RART pathway to promote PM delivery. We have shown that RalA, a fusion machinery component, is present on the R1 vesicle (Figure S2B), and exogenous expression of RalA-RED induced PM translocation of R1-GFP (Figure S2B and Figure 2E). Furthermore, endogenous RalA could be induced by Hx to co-localize with R1-GFP on the PM (Figure S5C, compare lower row with upper row). To confirm the role of RalA in mediating PM localization, we first demonstrated that PM localization of endogenous RalA could be enhanced after 24 h in SS or Nx conditions (arrows, Figure 5D, top row). Second, we showed that expression of constitutively active RalA-G23V forced PM localization of R1-GFP in Nx only in RalA-G23V expressing cells (asterisks, Figure 5D, middle row, arrow in inset) and dominant-negative RalA-S28N reduced PM translocation of R1-GFP in 24 h Hx (asterisks, Figure 5D, bottom row) and SS (asterisks, Figure S5D) only in RalA-S28N expressing cells, while non-expressing cells showed membrane localization of R1-GFP (sharp yellow arrows, Figure 5D, bottom row, and Figure S5D, lower row). From these results, we infer that RalA is a component of the RART pathway and RalA activity is required for PM translocation of FGFR1, likely at the fusion steps at the PM since RalA is known to be a membrane fusion factor.

Thus far our results indicate that PM localization is mechanistically linked to FDPS binding to FGFR1 C-terminus as the first step, and utilization of RalA activity is at the step of PM fusion. In order to determine the intermediary steps in the process, recruitment and activity of Rab proteins on the R1 and the ΔCR1 vesicles were examined in Nx, Hx and SS using HEK293-R1 and HEK293-ΔCR1 cells. The experimental results (summarized in Figure 5E) revealed three major points: (1) Rab2a is absent from the R1 vesicles or at low levels, but present on the ΔCR1 vesicles, in Hx and SS, and is present on both the R1 and the ΔCR1 vesicles in Nx; (2) Rab6a is present on the R1, but not on the ΔCR1 vesicles, in Nx, Hx and SS; (3) the ΔCR1 vesicles contain significantly lower Rab8a; and (4) RalA is a constitutive component of the R1 vesicles therefore suggesting that it may be activated only in the presence of other Rab proteins. The requirement of Rab8a in RART pathway was further supported by our observation that constitutively active Rab8a(Q67L) stimulated PM localization of R1-GFP in Nx (Figure 5F) and siRNA-mediated depletion of Rab8a reduced R1-GFP cell surface levels in SS at 24 h, using surface biotinylation assay (Figure S6A, compare lanes 3 and 4 with lanes 1 and 2). The observed Rab dynamics on the R1 vesicles (summarized in Figure 5E) suggests that specific Rab activity is selectively utilized on the R1 vesicles to regulate the sequential steps necessary for PM delivery.

**Figure 6:**
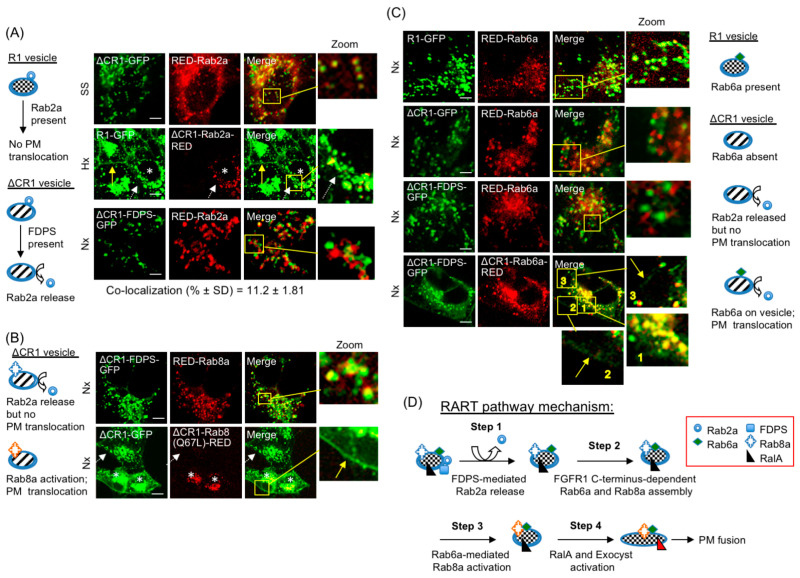
Identification of regulatory ON/OFF switches of the RART pathway. The schematics on left and right of image panels show ON/OFF switches of the RART pathway. Note: since RED is fused to C-terminus of Rab proteins, their recruitment to the R1 vesicle is CaaX-independent and is dependent on localization as ΔCR1 fusions. (**A**) The inhibitory effect of Rab2a on PM localization of R1-GFP was studied using ΔCR1-Rab2a-RED fusion. The top row shows that in the absence of FGFR1 C-terminus in ΔCR1-GFP, Rab2a is not released from the R1 vesicles in 24 h SS. In second row, cells expressing ΔCR1-Rab2a-RED (asterisks) show no PM translocation of R1-GFP in 24 h Hx (dashed white arrow), while neighboring cells show R1-GFP on the PM (yellow arrow). The third row shows that Rab2a is released in Nx upon direct recruitment of FDPS to the R1 vesicle as ΔCR1-FDPS-GFP chimera. (**B**) Release of Rab2a using ΔCR1-FDPS-GFP failed to promote PM translocation even when Rab8a level was increased by co-expressing RED-Rab8a (top row). In the bottom row, direct recruitment of constitutively active Rab8a to the R1 vesicle in cells expressing ΔCR1-Rab8a(Q67L)-RED (asterisks), led to presence of ΔCR1-GFP on the PM (yellow arrow in inset), while neighboring untransfected cells show no PM localization in Nx (dashed white arrow), showing that Rab8a activation on the R1 vesicle is necessary to stimulate PM translocation. (**C**) RED-Rab6a is present in the R1 vesicles (top row), but not in the ΔCR1 vesicles (second row). Release of inhibitory Rab2a using ΔCR1-FDPS-GFP failed to recruit RED-Rab6a to the ΔCR1 vesicles (third row). Three insets are shown in enlarged views. Vesicle localization of Rab6a using ΔCR1-Rab6a-RED (inset 1 in the bottom row), accompanied by release of Rab2a using ΔCR1-FDPS-GFP, led to detectable increase in PM localization of ΔCR1-FDPS-GFP (bottom row, arrows in insets 2 and 3). (**D**) The model for regulatory ON/OFF switches in the RART pathway and their activation sequence is presented. Yellow boxes are regions of interest that are enlarged (Zoom). Bars are 20 μm.

To determine the sequential order of Rab activities leading to PM delivery, specific Rab proteins of the RART pathway were localized to the R1 vesicle in lipidation-independent manner by blocking their C-terminus with RED and directing their localization/activity as ΔCR1 chimeras (Figure S6B), a novel strategy we successfully employed earlier to localize FDPS to the R1 vesicles (see above). Since RED is fused to C-terminus of Rab proteins, their recruitment to the R1 vesicle is CaaX-independent and is dependent on localization as ΔCR1 fusions. Such ΔCR1-Rab-RED fusions were expressed in HEK293-R1 or HEK293-ΔCR1 cells to turn on/off PM delivery as detailed below. Similar results were obtained in SS and Hx; therefore, only representative results are discussed in the following sections to avoid presentation of voluminous data. RED-Rab2a was not released from the ΔCR1 vesicles in HEK293-ΔCR1 cells at 24 h SS (Figure 6A, top row and Figure 5E). Therefore, involvement of Rab2a as a negative regulator of PM translocation was suspected. To confirm this, ΔCR1-Rab2a-RED was expressed in HEK293-R1. The ΔCR1-Rab2a-RED chimera localized to the R1 vesicles and led to inhibition of PM localization of R1-GFP in Hx at 24 h (asterisk, Figure 6A, second row), confirming Rab2a as a negative switch on the R1 vesicles. This is also consistent with the finding that Rab2a is greatly reduced in the R1 vesicle (which is PM-bound) in SS and Hx, but persists on the ΔCR1 vesicles (which are not PM-bound) in the same conditions (Figure 5E).

To determine how the inhibitory Rab2a is removed from the R1 vesicle, ΔCR1-FDPS-GFP chimera was expressed to localize FDPS to the R1 vesicle. Expression of ΔCR1-FDPS-GFP released RED-Rab2a in Nx (Figure 6A, third row), confirming that FDPS binding to FGFR1 on the R1 vesicle leads to removal of the inhibitory Rab2a from the R1 vesicles. However, in Nx, Rab2a release alone failed to induce PM localization of ΔCR1-FDPS-GFP, a fusion that lacks the full FGFR1 C-terminus (Figure 6A, third row). We infer that Rab2a release alone is insufficient for FGFR1 PM delivery.

Next, using ΔCR1-FDPS-GFP, we tested if increased Rab8a level could stimulate PM localization following Rab2a release by FDPS. Our results (Figure 6B, top row) showed that, although Rab2a release as a result of ΔCR1-FDPS-GFP expression (Figure 6A, third row) and increased RED-Rab8a localization to the R1 vesicles (Figure 6B, top row) occurred, the ΔCR1-FDPS-GFP chimera did not localize to the PM in Nx. On the other hand, when ΔCR1-Rab8a(Q67L)-RED chimera was expressed in Nx to localize the constitutively active form of Rab8a directly to ΔCR1-GFP vesicles, PM translocation of ΔCR1-GFP was detectable even without expression of ΔCR1-FDPS-GFP, presumably without requiring Rab2a release, because the ΔCR1-GFP vesicles contain neither FDPS nor the full FGFR1 C-terminus (Figure 6B, second row). The result indicates that Rab8a activation is the key regulatory step that functions downstream of FDPS and Rab2a, and FGFR1 C-terminus functions as a scaffold for assembly of factors necessary for Rab8a activation. The collective evidence presented above suggests that Rab8a activation on the R1 vesicle is a rate-limiting step in the PM translocation of FGFR1.

What activates Rab8a? Rab6a is present on the R1 vesicles (Figure 5E and Figure 6C, top row) but is absent from the ΔCR1 vesicles, especially in the cell periphery (Figure 5E and Figure 6C, second row). It has been shown that Rab6 is needed for Rab8a function in secretory vesicle docking and exocytosis [56]. However, secretory vesicle exocytosis utilizes a distinct mechanism and occurs with faster kinetics compared to that observed for the R1 vesicle. The fact that release of Rab2a, by expressing ΔCR1-FDPS-GFP (Figure 6A, third row), did not lead to extensive localization of RED-Rab6a to RART vesicle (Figure 6C, third row) served as further confirmation that FGFR1 full C-terminus is required for Rab6a recruitment and that Rab2a release alone is insufficient. In light of the absence of Rab6a on the ΔCR1 vesicle, we tested if the functional link between Rab6a and Rab8a is relevant to the RART pathway. For this, we took advantage of a low but detectable Rab8a level on the ΔCR1 vesicle to determine if Rab6a acted upstream of Rab8a. If Rab6a acted upstream of Rab8a, meaning if Rab6a activates Rab8a, weak PM localization would occur when the Rab6a level is increased on the ΔCR1 vesicles because the low Rab8a level on the ΔCR1 vesicles is the rate-limiting factor, as discussed above. Rab6a was designed as ΔCR1-Rab6a-RED chimera and co-expressed with ΔCR1-FDPS-GFP, which we have shown to release Rab2a (Figure 6A, third row). In this experiment performed in Nx, ΔCR1-FDPS-GFP is expected to release Rab2a and ΔCR1-Rab6a-RED is expected to activate Rab8a. Remarkably, although the effect was weak as we predicted, due to low Rab8a level on the ΔCR1 vesicle, PM translocation of ΔCR1-FDPS-GFP was detectable (Figure 6C, bottom row, arrows in insets), providing the direct evidence that Rab6a mediates Rab8a activation on the R1 vesicle. From the combined data presented above we infer that FGFR1 C-terminus is a scaffold for binding FDPS and facilitating Rab6a-Rab8a assembly and activation, leading to PM delivery. In summary, two distinct domains in FGFR1 C-terminus participate in defined steps of PM delivery; i.e., domain-I (aa 398–580) is involved in FDPS binding and subsequent Rab2a release, while domain-II (aa 581–822) facilitates Rab6a/Rab8a activation on the R1 vesicle.

Based on our results, we propose a mechanistic model for the RART pathway (Figure 6D). Conditions in tissue microenvironment such as hypoxia or nutrient starvation trigger sequential activation steps, with FGFR1 C-terminus functioning as scaffold for assembly and activation of the regulatory switches. Rab2a release from the R1 vesicles follows FDPS binding to FGFR1 C-terminus domain-I, leading to functional assembly of Rab6a and Rab8a by domain-II of FGFR1 C-terminus and localized Rab6a-mediated activation of Rab8a. Downstream of Rab8a activation, our results suggest that activation of RalA occurs to deliver FGFR1 to the cell surface via kiss-and-run partial PM fusion. Note that recycling vesicles predominantly undergo full fusion with the PM (unlike kiss-and-run fusion) [34]. The precise timing of RalA activation requires further study to understand its temporal relationship to Rab6a/Rab8a activation and the final PM fusion step. Our observations suggest tubular extensions of the R1 vesicle (Figure 3D,E) could allow partitioning of fusion-competent FGFR1 scaffold to vesicular sub-domains, which is mechanistically compatible with both the observed exclusion of ΔCR1-GFP on the PM and the kiss-and-run mode of delivery for PM fusion. The kiss-and-run fusion has been observed to require tethering to an exocyst for a longer duration to enable cargo delivery at the PM [53], consistent with our live cell observations. Note that the RART pathway is reversible and the components revert to FGFR1 sequestration mode upon return of Nx and normal nutritional status.

### 3.10. The RART Pathway’s Role in mEB Development

Next, we examined the biological relevance of the RART pathway using mouse embryoid bodies (mEBs). mEB is an aggregate of mouse embryonic stem cells (mESCs), which replicates early embryonic development in vitro, such as formation of neurons, pancreatic islets and cardiomyocytes [41]. FGFR1/2 signaling is essential for coronary vasculature [57] and heart development [58]. Such differentiated mEBs will ‘beat’ under appropriate conditions even without a fully developed heart [59]. Cardiomyocyte differentiation and ‘beating’ phenotype are linked to the FGFR activities [22,60], with *Fgfr1* expression starting early at day 0, while *Fgfr2-4* expression begins around day 7 of mEB development [60]. Therefore, the mEB system is convenient for temporally isolating the activity of FGFR1 from the activities of other FGF receptors, FGFR2-4.

**Figure 7:**
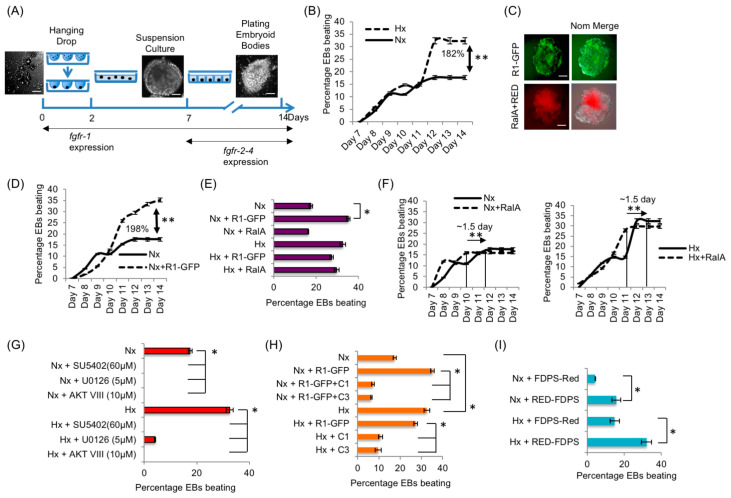
The RART pathway operates in FGFR1-mediated early embryonic development. (**A**) Schematic showing EB culture period of 14 days and the corresponding known *Fgfr* expression pattern. (**B**) Hx resulted in 182% higher percentage of beating EBs compared to Nx. (**C**) Typically, 80% transfection efficiency was achieved in EBs, demonstrated by transfection of plasmid expressing R1-GFP (green) and co-transfection of RalA and RED (red; Nom. = Nomarski optics). (**D**) Transfection of R1-GFP into mESC at day 2 significantly enhanced EB beating by ~twofold (198%) in Nx. (**E**) R1-GFP expression increased the percentage beating significantly in Nx, but could not increase beating further in Hx. On the other hand, transfection of RalA did not increase percentage EB beating in Nx or Hx. (**F**) Although exogenous RalA expression did not increase the percentage of EB beating (shown in **E**), EB beating reaches maximum capacity by 1.5 days earlier in both Nx (left panel) and Hx (right panel) upon RalA overexpression. (**G**) FGFR1 inhibitor (SU5402) or inhibitor of its downstream signaling components (U0126) inhibited EB beating nearly completely in Nx and Hx. (**H**) Consistent with the results shown in Figure 5B, expression of C1-RED and C3-RED inhibited EB beating by ~65–75% in Nx and Hx. (**I**) Expression of cytosolic RED-FDPS enhanced EB beating in both Nx and Hx, as predicted. * *p* < 0.05 and ** *p* < 0.01. The n value for each experimental set of EB analysis is provided in Section 2.12. Bars are 100 μm.

mEB beating phenotype was scored for 14 days (Figure 7A). mEB differentiation in the presence of Hx condition was confirmed by the decrease in expression of stem cell marker stage-specific embryonic antigen-1 (SSEA-1; Figure S7). The expression of endogenous FGFR1 was also confirmed (Figure S7). A 1.8-fold increase in the percentage of beating mEBs was observed in Hx compared with Nx (Figure 7B). In the mEB experiments, plasmid DNA transfection efficiency consistently approached 80%, as judged by GFP/RED fluorescence (Figure 7C). Collective results in the mEB experiments successfully recapitulated the primary observations of the RART pathway in HEK293 cell cultures: first, exogenous R1-GFP expression increased mEB beating by 198% in Nx (Figure 7D) but not in the induced state of Hx (Figure 7E). Second, exogenous RalA expression did not lead to measurable enhancement in beating (Figure 7E), but mEB beating reached maximum ~1.5-days earlier when RalA is expressed in either Nx or Hx (Figure 7F). Third, inhibitors of FGFR1 (SU5402) and Erk1/2 (U0126) blocked mEB beating, linking the beating phenotype to FGFR1 activity (Figure 7G), consistent with published reports. Fourth, co-expression of R1-GFP with C1 or C3 peptides (Figure 5B) inhibited the increased beating observed when R1-GFP alone was expressed in an mEB model in Nx (Figure 7H). Similarly, C1 and C3 expression inhibited beating in Hx (Figure 7H). Note that C3 peptide only partially inhibited FGFR1 PM localization (Figure 5B) but could completely inhibit mEB beating. This is possibly because mEB beating is more sensitive to changes in FGFR signaling strength. Finally, forced cytosolic localization of FDPS, by expressing RED-FDPS, enhanced the mEB beating significantly in Nx and Hx (Figure 7I). The mEB model, which utilizes FGFR1 signaling activity beginning at very early stages, clearly demonstrated the relevance of the RART pathway to critical developmental outcomes.

## 4. Discussion

Among others, PM-localized proteins span the diverse spectrum of RTKs, cell–cell and cell–matrix adhesion proteins, growth hormone receptors, G-protein coupled receptors (GPCRs) and those involved in immune regulation. Previous observations noted the localization of leptin receptor and FGFR3 in intracellular vesicles, and vascular endothelial growth factor receptor-2 (VEGFR2) in Golgi [42,61,62]. While the observed FGFR3 intracellular localization remains uncharacterized, ER-associated RNF121 activity increases cell surface localization of Golgi-localized VEGFR2 by reducing ubiquitination and promoting maturation [63]. On the other hand, mutations that disrupt anterograde trafficking can result in intracellular retention of surface receptors and are directly linked to disease pathogenesis, such as those in anaplastic lymphoma kinase (ALK), insulin-like growth factor 1 receptor (IGF1R), melanocortin 1 receptor (MC1R), bone morphogenic protein type-II receptor (BMPR-II) and the potassium channel Kir2.1 [64,65,66,67,68].

Prior studies, including ours, detected FGFR1 IIIc in intracellular vesicles in a variety of cells and tissues under normal conditions [14,23,69], but its relevance to FGFR1 signaling remained unexplored. We present evidence showing that FGFR1 is sequestered in the RART vesicles, which we characterize as positive for Rab2a, Rab6a, Rab8a, caveolins and RalA, resulting in the inhibition of FGFR1 signaling. Thus, these RART vesicles carry a unique combination of GTPases that are distinct from known endocytic or recycling vesicles. Our current results clearly distinguish the RART pathway as inherently different from the vectoral or transcytotic routes because vesicle-sequestered FGFR1 constitutes a steady-state pool of this protein. Our experiments also show that the RART pathway differs from regulated non-secretory exocytosis. First, the pathway operates with slow kinetics. Second, PM delivery does not involve full vesicle fusion. Therefore, the RART pathway represents the first example of a regulated RTK sequestration mechanism.

Our results further identify the protein FDPS as a key organizer of the GTPases involved in the RART vesicle PM targeting. FDPS may function as an adaptor such as the Naked-2 protein in basolateral sorting of transforming growth factor-alpha [70]. Interestingly, an in vivo link to signaling and primordial germ cell migration through the FDPS pathway has been demonstrated previously in *Drosophila* [71]. The precise mechanism by which FDPS interaction with the FGFR1 domain-I results in release of Rab2a requires further work. FDPS could function as a non-conventional adaptor, which replaces and attracts different GTPases, or localized FDPS enzymatic activity could modify and recruit specific GTPases, including Rab proteins. It also remains to be seen if FDPS-stimulated cell surface localization regulates yet other RTK(s), similar to our observation with FGFR1 in this study.

Importantly, we observe that the domain-II of FGFR1 directly stabilizes Rab6a/Rab8a on the RART vesicle. siRNA-mediated depletion of Rab8a led to markedly reduced surface localization of FGFR1. Engagement of RalA-exocysts and Rab8a-exocysts during the final steps of PM fusion is known [72,73,74]. It is possible that Rab8a brings Rab6a in proximity with effector proteins such as RalA and microtubule-associated motors, and directs the vesicles toward the PM fusion sites to integrate microtubule dynamics with kiss-and-run fusion.

Indeed, in this report, we demonstrate the requirement of RalA activity, but not RalB, on the RART vesicles (Figure S2B). This supports the conclusion that Rab6a-Rab8a-RalA cooperates functionally during PM delivery, and reaffirms the isoform-specific roles played by RalA and RalB in diverse cellular processes [75]. Caveolin 1 and 2 are also present on the RART vesicles (Figure 5E). Their exact roles in the RART pathway need further studies.

In this study, we find that the RART pathway ON/OFF switches are functionally relevant in FGFR1-mediated mEB development. Low fetal oxygen microenvironment is essential for embryonic development and normal pulmonary development takes place in hypoxic environment of the uterus [76,77]. Hypoxia also plays significant roles in kidney and cardiovascular organogenesis [78,79,80]. FGFR1 signaling, coupled with hypoxia and morphogen waves, are known to be critical for gradual patterning of these organs’ development and the slow PM accumulation kinetics of FGFR1 observed for the RART pathway is compatible with these in vivo developmental timescales. We have shown that through FDPS-FGFR1 interaction, the RART pathway has the intrinsic capability to respond to tissue microenvironmental cues, at least through the subcellular localization of FDPS (ER vs. cytosol). Such a mechanism is critical during normal development, and likely is exploited in solid tumor growth. The interplay between hypoxia and FGFR1 is arguably best evidenced during guided branching morphogenesis of the *Drosophila* tracheal system [51]. In this system, clusters of hypoxic cells secrete Branchless, the *Drosophila* FGF-ligand, to attract tracheal cells that express Breathless (*Drosophila* FGFR homolog), and this functional interplay ensures construction of the tracheal network in step to match tissue oxygen demand. Breathless/FGFR is presumed to be constitutively present on the tracheal cell surface, awaiting stimulation by Branchless/FGF to induce tracheal cell migration towards increasing FGF ligand gradient. Identification of the novel RART pathway offers the possibility that tracheal cells could translocate FGFR1 to the cell surface “on-demand” to fine-tune the tracheal cell migration response.

It is difficult to predict the existence of mechanisms similar to the RART pathway for other RTKs. Given that FGFR3 is seen predominantly in intracellular vesicles under normal conditions, it is worth investigating this phenomenon in the context of RART and FGFR signaling pathways. On the other hand, based on the known utilization of spatially and temporally integrated signaling outputs from multiple RTKs to achieve directed cell migration [81,82,83,84], we speculate that neuronal path-finding and vascular development could involve variation(s) of the RART pathway.

In neurons, the neurotrophin receptors, Tropomyosin-related kinase A, B and C (TrkA, TrkB and TrkC), are synthesized in the cell body and transported to axonal PM over long distances. Interestingly, TrkB has been shown to interact with Rab8a in co-immunoprecipitation studies and knockdown of Rab8a also reduced PM localization of TrkB, without increasing internalization of TrkB [85]. Rab8 is present in axons and dendrites of neurons at early developmental stages and antisense oligonucleotide-mediated reduction in Rab8 expression inhibits membrane traffic and blocks maturation of neurons [86]. It is also known that TrkB is trafficked directly to axonal PM via Rab6 carrier secretory vesicles [87], and the cytoplasmic portion of TrkB serves as a scaffold for assembly of Rab27, collapsin response mediator protein-2 (CRIMP-2) and Exophilin-7, with CRIPM-2 linking to the microtubule motor protein Kinesin-1 to accomplish microtubule-based anterograde transport followed by complete vesicle fusion with axonal PM [88]. As such, the TrkB PM targeting may involve a mechanism similar to that of the RART pathway, with the difference in complete vs. partial vesicle fusion. However, the interplay, if any, between Rab8a, Rab6 and Rab27 is not yet clear with respect to TrkB anterograde transport. In contrast, TrkA is initially delivered to somatodendritic membranes in a non-targeted manner followed by endocytosis and re-targeting to axonal membrane through the mechanism of vectoral transcytosis [89] involving Rab11 recycling endosomes and complete vesicle fusion at the re-targeted PM. TrkA and TrkB’s axonal membrane localization/re-targeting is reinforced in the direction of the PM domains closest to the source of the neurotrophin ligand, thereby strengthening neurotrophin-mediated signaling, influencing axonal growth and survival. It is not immediately evident from our study if such a ligand-directed targeting/re-targeting of FGFR1 to distinct PM domains is an integral part of the RART pathway mechanism, but RART pathway does not involve complete vesicle fusion, which is the typical mechanism of secretory and recycling vesicle fusion to the PM.

Based on our study, the RART pathway confers the cellular ability to respond to microenvironmental cues to selectively utilize FGFR1 to fine-tune signaling output from the FGFR-FGF system, a mechanism that could be critical when multiple high-affinity receptor-ligand paring is possible among the four major receptors (FGFR1-4), their isoforms and the 23 known FGF ligands.

## 5. Conclusions

FGFR1 IIIc is sequestered and actively prevented from PM localization under normal conditions. We identify the regulatory switches that sequester FGFR1 and the mechanism activated for its PM localization. This is the first instance of an RTK sequestered intracellularly for regulated PM delivery. It is highly likely that the RART pathway dynamics play a significant role in cell behavior of solid tumors, since hypoxia and limiting nutritional state are universally applicable to all solid tumors. Therefore, identifying therapeutic targets within the RART pathway, as a means to block FGFR1 PM localization and activation, is a plausible approach.

## Data Availability

All data generated or analyzed during this study are included in this published article and its supplementary information files. All materials generated in this study are available to academic researchers upon request.

## Supplementary Materials

The following supporting information can be downloaded at: https://www.mdpi.com/article/10.3390/cancers15245837/s1, Figure S1: Kiss-and-run movement of the FGFR1 vesicle to the plasma membrane. Figure S2. Selective presence of GTPase to the FGFR1 vesicles. Figure S3. Vesicle-localized FGFR1 represents a stable pool. Figure S4. FGFR1 activation determines cell motility. Figure S5. C-terminus of FGFR1 and RalA activity are required for FGFR1 membrane localization under starvation and hypoxia. Figure S6. Rab8a activity is required for FGFR1 PM localization. Figure S7. Hypoxia induces FGFR1 surface localization and differentiation of mouse embryoid bodies (EBs). Video S1. The R1 vesicles projected tubular extensions and exhibited curvilinear motion. Video S2. Single-vesicle tracking of the R1 vesicles. Video S3. Kiss-and-run transfer of the R1 vesicles undergoing PM fusion. Video S4. PM delivery of the FGFR1 vesicle in hypoxia after cycloheximide block. Video S5. PM delivery of the FGFR1 vesicle in serum starvation after cycloheximide block.

## Abbreviations

ALK	Anaplastic lymphoma kinase
ATCC	American Type Culture Collection
AMPA	α-amino-3-hydroxy-5-methyl-4-isoxazolepropionic acid
AQP-2	Aquaporin-2
BMPR-II	Bone morphogenic protein type-II receptor
CLASP	Cytoplasmic linker-associated protein
CHX	Cycloheximide
DHSB	Developmental studies hybridoma bank
EGFP	Enhanced green fluorescence protein
ENACs	Epithelial sodium channels
EMT	Epithelial to mesenchymal transition
Erk1/2	Extracellular signal-regulated kinase 1/2
FDPS/FPPS	Farnesyl diphosphate synthase
FGFR1	Fibroblast growth factor receptor-1
FGFs	Fibroblast growth factors
GPCRs	G-protein coupled receptors
GLUT4	Glucose transporter 4
hVHL	Human von-Hippel Lindau
Hx	Hypoxia
IGF1R	Insulin-like growth factor 1 receptor
MC1R	Melanocortin 1 receptor
MICAL3	Microtubule-associated monooxygenase, calponin and LIM Domain Containing 3
mEBs	Mouse embryoid bodies
Nx	Normoxia
PM	Plasma membrane
PGVs	Post-Golgi vesicles
ELKS	Protein that is rich in amino acids E, L, K and S
RTK	Receptor tyrosine kinase
RART	Regulated anterograde RTK transport
siRNA	Small interfering RNA
SS	Serum starvation
SSEA-1	Stage specific embryonic antigen-1
TIRFM	Total internal reflection fluorescence microscopy
TGF-α	Transforming growth factor-alpha
VEGFR2	Vascular endothelial growth factor receptor-2
