# Supplementary material for: Controlled Plasma Membrane Delivery of FGFR1 and Modulation of Signaling by a Novel Regulated Anterograde RTK Transport Pathway
Source: Cancers (Basel). 2023 Dec 14;15(24):5837. doi: 10.3390/cancers15245837 (PMC10741464; doi:10.3390/cancers15245837)
Supplement: Supplementary file 1 [file cancers-15-05837-s001.zip › Dammai-Claire-Suppfigs(RART-Cancers-Final)-7.pptx]

## Slide 1
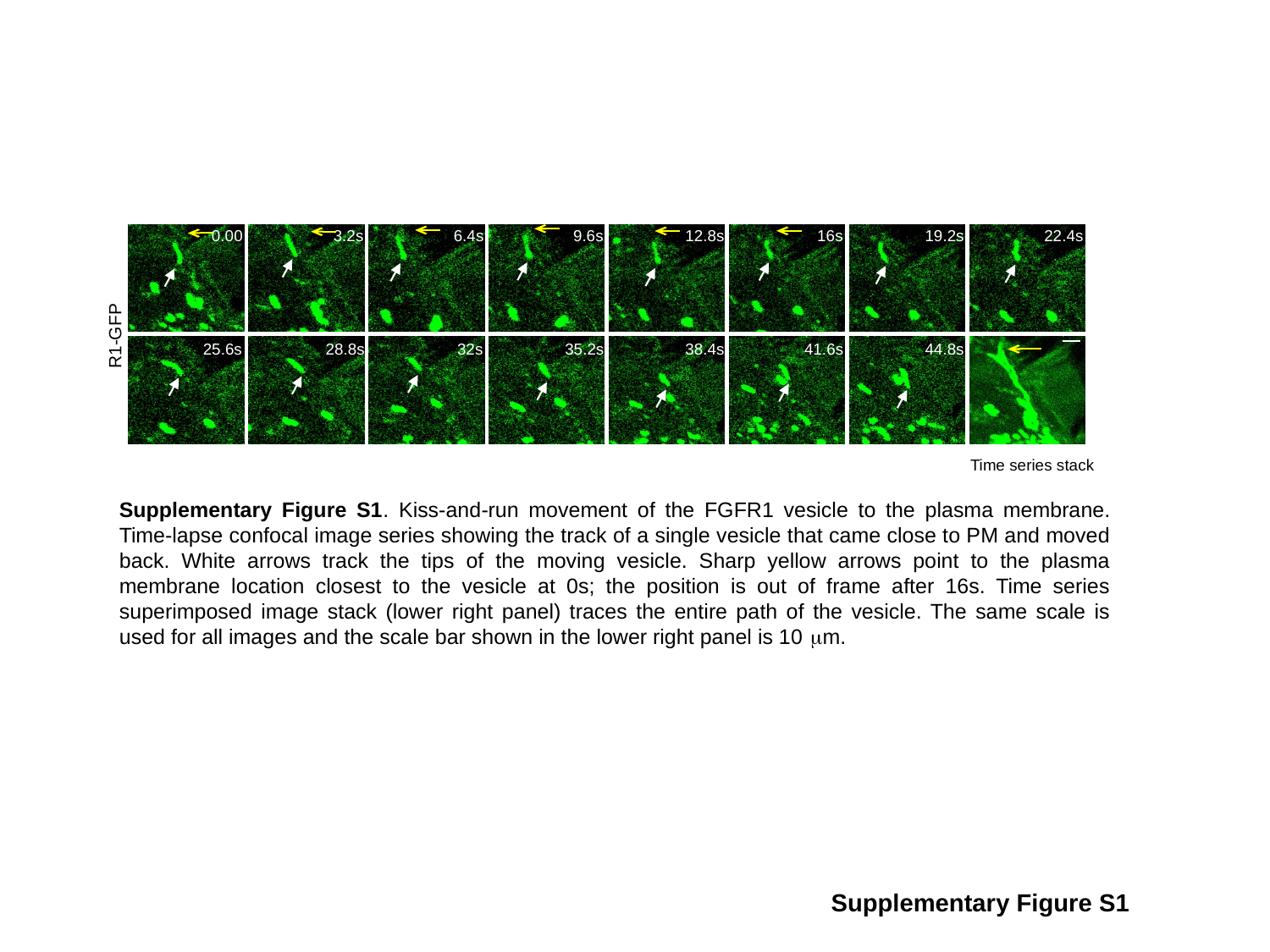

12.8s
19.2s
9.6s
22.4s
6.4s
16s
0.00
3.2s
R1-GFP
28.8s
35.2s
41.6s
44.8s
38.4s
32s
25.6s
Time series stack
Supplementary Figure S1. Kiss-and-run movement of the FGFR1 vesicle to the plasma membrane. Time-lapse confocal image series showing the track of a single vesicle that came close to PM and moved back. White arrows track the tips of the moving vesicle. Sharp yellow arrows point to the plasma membrane location closest to the vesicle at 0s; the position is out of frame after 16s. Time series superimposed image stack (lower right panel) traces the entire path of the vesicle. The same scale is used for all images and the scale bar shown in the lower right panel is 10 mm.
Supplementary Figure S1

## Slide 2
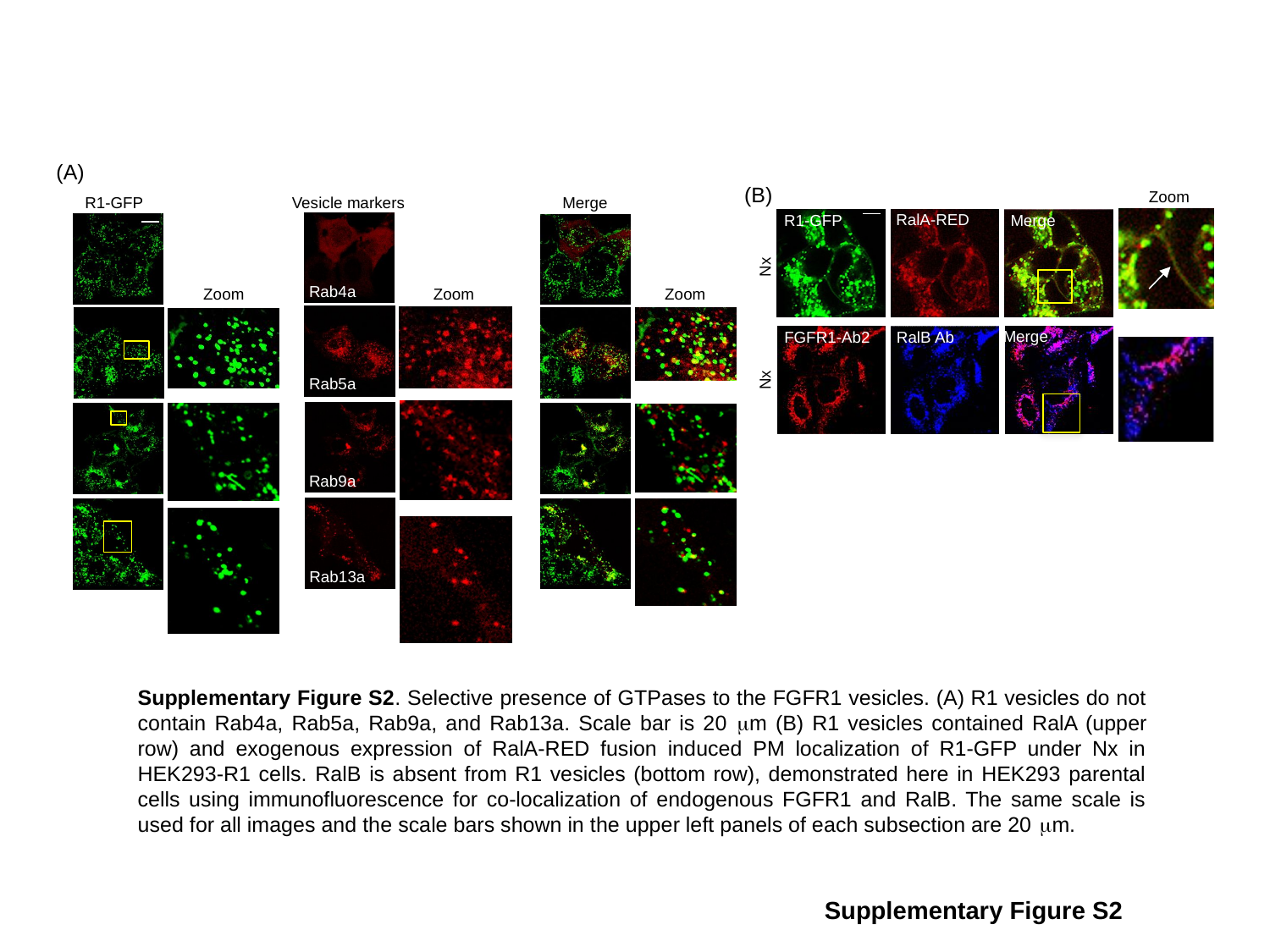

(A)
R1-GFP
Vesicle markers
Merge
Rab4a
Zoom
Zoom
Zoom
Rab5a
Cav1-RFP
Rab9a
Rab13a
(B)
Zoom
RalA-RED
Merge
R1-GFP
Nx
Merge
FGFR1-Ab2
RalB Ab
Nx
Supplementary Figure S2. Selective presence of GTPases to the FGFR1 vesicles. (A) R1 vesicles do not contain Rab4a, Rab5a, Rab9a, and Rab13a. Scale bar is 20 mm (B) R1 vesicles contained RalA (upper row) and exogenous expression of RalA-RED fusion induced PM localization of R1-GFP under Nx in HEK293-R1 cells. RalB is absent from R1 vesicles (bottom row), demonstrated here in HEK293 parental cells using immunofluorescence for co-localization of endogenous FGFR1 and RalB. The same scale is used for all images and the scale bars shown in the upper left panels of each subsection are 20 mm.
Supplementary Figure S2

## Slide 3
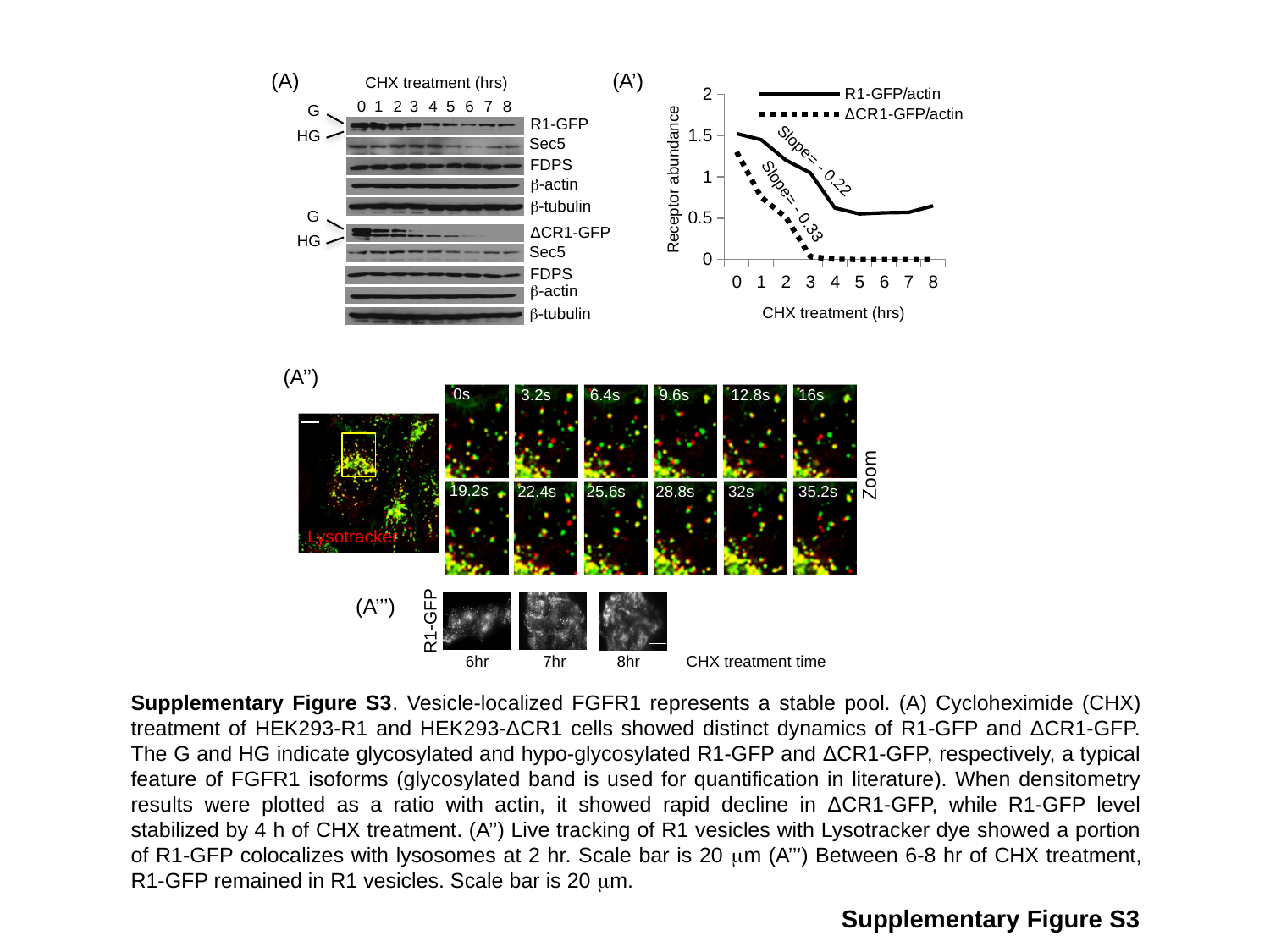

(A)
(A’)
CHX treatment (hrs)
### Chart
| Category | R1-GFP/actin | ΔCR1-GFP/actin |
|---|---|---|
| 0 | 1.526 | 1.304 |
| 1 | 1.451 | 0.754 |
| 2 | 1.205 | 0.513 |
| 3 | 1.05 | 0.035 |
| 4 | 0.624 | 0.004 |
| 5 | 0.553 | 0.0 |
| 6 | 0.566 | 0.0 |
| 7 | 0.571 | 0.0 |
| 8 | 0.65 | 0.0 |Slope= - 0.22
Receptor abundance
Slope= - 0.33
CHX treatment (hrs)
0
1
2
3
4
5
6
7
8
G
R1-GFP
HG
Sec5
FDPS
b-actin
b-tubulin
G
ΔCR1-GFP
HG
Sec5
FDPS
b-actin
b-tubulin
(A’’)
0s
3.2s
6.4s
9.6s
12.8s
16s
19.2s
22.4s
25.6s
28.8s
32s
35.2s
Lysotracker
(A’’’)
R1-GFP
6hr
7hr
8hr
CHX treatment time
Zoom
Supplementary Figure S3. Vesicle-localized FGFR1 represents a stable pool. (A) Cycloheximide (CHX) treatment of HEK293-R1 and HEK293-ΔCR1 cells showed distinct dynamics of R1-GFP and ΔCR1-GFP. The G and HG indicate glycosylated and hypo-glycosylated R1-GFP and ΔCR1-GFP, respectively, a typical feature of FGFR1 isoforms (glycosylated band is used for quantification in literature). When densitometry results were plotted as a ratio with actin, it showed rapid decline in ΔCR1-GFP, while R1-GFP level stabilized by 4 h of CHX treatment. (A’’) Live tracking of R1 vesicles with Lysotracker dye showed a portion of R1-GFP colocalizes with lysosomes at 2 hr. Scale bar is 20 mm (A’’’) Between 6-8 hr of CHX treatment, R1-GFP remained in R1 vesicles. Scale bar is 20 mm.
Supplementary Figure S3

## Slide 4
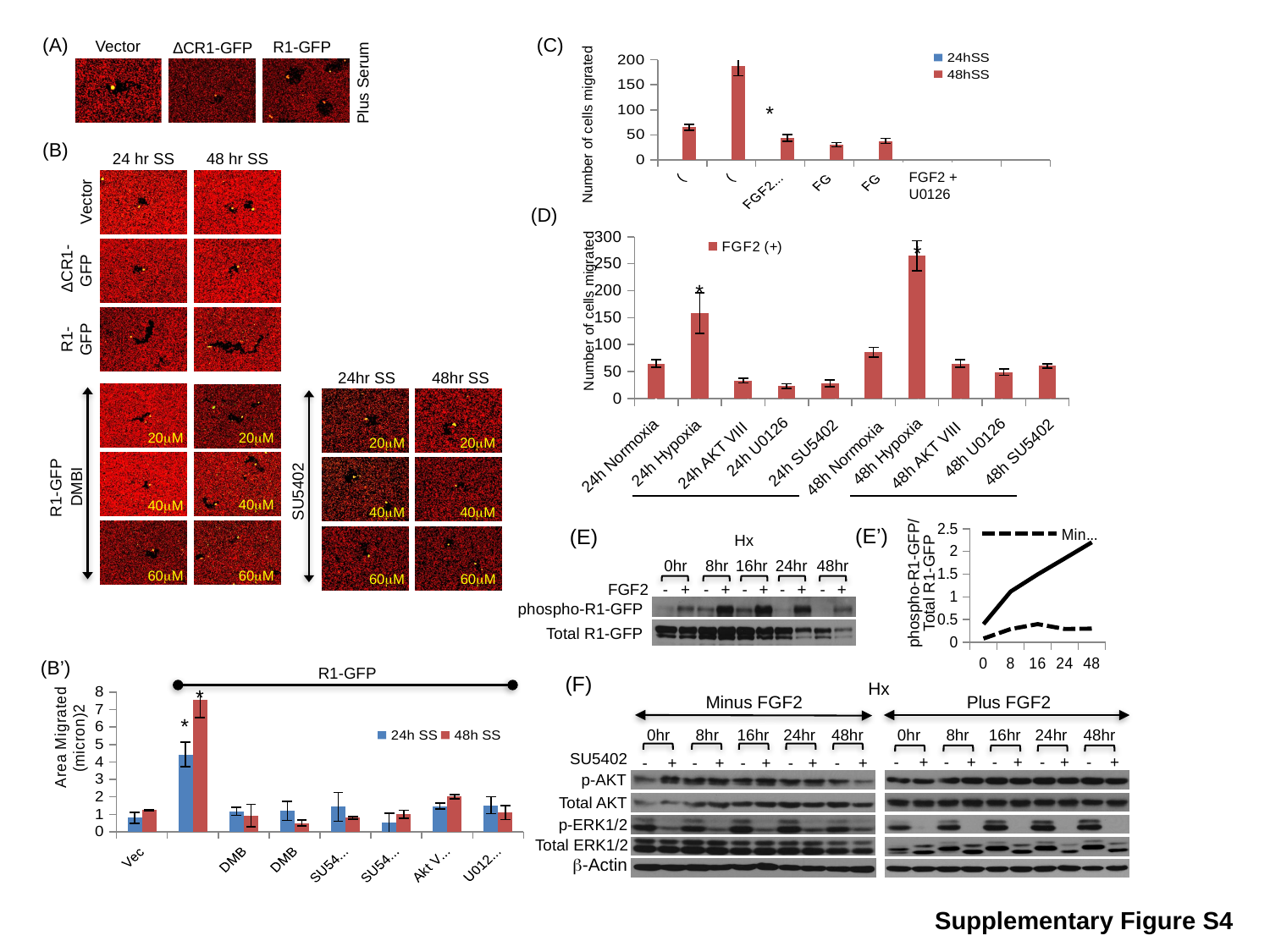

### Chart
| Category | 24hSS | 48hSS |
|---|---|---|
| (-) FGF2 | 38.0 | 65.0 |
| (+) FGF2 | 79.5 | 187.5 |
| FGF2 + AKTVIII | 26.0 | 43.75 |
| FGF2 + U0216 | 21.5 | 30.0 |
| FGF2 + SU5402 | 31.25 | 37.75 | Number of cells migrated
FGF2 +
U0126
(C)
(A)
Vector
R1-GFP
∆CR1-GFP
Plus Serum
*
(B)
48 hr SS
24 hr SS
### Chart
| Category | FGF2 (+) |
|---|---|
| 24h Normoxia | 65.0 |
| 24h Hypoxia | 158.3 |
| 24h AKT lnhib | 33.25 |
| 24h ERK Inhib | 23.0 |
| 24h SU5402 | 28.25 |
| 48h Normoxia | 86.0 |
| 48h Hypoxia | 264.8 |
| 48h AKT lnhib | 65.0 |
| 48h ERK Inhib | 48.75 |
| 48h SU5402 | 60.5 |
Vector
(D)
*
 ∆CR1-
GFP
*
R1-
GFP
48hr SS
24hr SS
20mM
20mM
20mM
20mM
24h U0126
48h U0126
48h Hypoxia
48h SU5402
24h Hypoxia
24h SU5402
48h AKT VIII
24h AKT VIII
24h Normoxia
48h Normoxia
DMBI
R1-GFP
SU5402
40mM
40mM
40mM
40mM
(E’)
### Chart
| Category | Minus FGF2 | Plus FGF2 |
|---|---|---|
| 0 | 0.08 | 0.397 |
| 8 | 0.289 | 1.115 |
| 16 | 0.398 | 1.494 |
| 24 | 0.291 | 1.845 |
| 48 | 0.299 | 2.2 |phospho-R1-GFP/
Total R1-GFP
(E)
Hx
0hr
8hr
16hr
24hr
48hr
-
FGF2
-
+
-
+
-
+
+
-
+
phospho-R1-GFP
Total R1-GFP
60mM
60mM
60mM
60mM
(B’)
R1-GFP
(F)
Hx
Minus FGF2
Plus FGF2
0hr
8hr
16hr
24hr
48hr
0hr
8hr
16hr
24hr
48hr
SU5402
-
+
-
+
-
+
-
+
-
+
-
+
-
+
-
+
-
+
-
+
p-AKT
Total AKT
p-ERK1/2
Total ERK1/2
b-Actin
*
### Chart
| Category | 24h SS | 48h SS |
|---|---|---|
| Vector | 0.8 | 1.24 |
| DMSO | 4.42 | 7.53 |
| DMBI-40µM | 1.18 | 0.93 |
| DMBI-60µM | 1.19 | 0.5 |
| SU5402-40µM | 1.427999999999999 | 0.8 |
| SU5402-60µM | 0.527 | 1.0 |
| Akt VIII-10µM | 1.47275 | 2.015 |
| U0126 -5µM | 1.519725 | 1.10424 |*
Supplementary Figure S4

## Slide 5
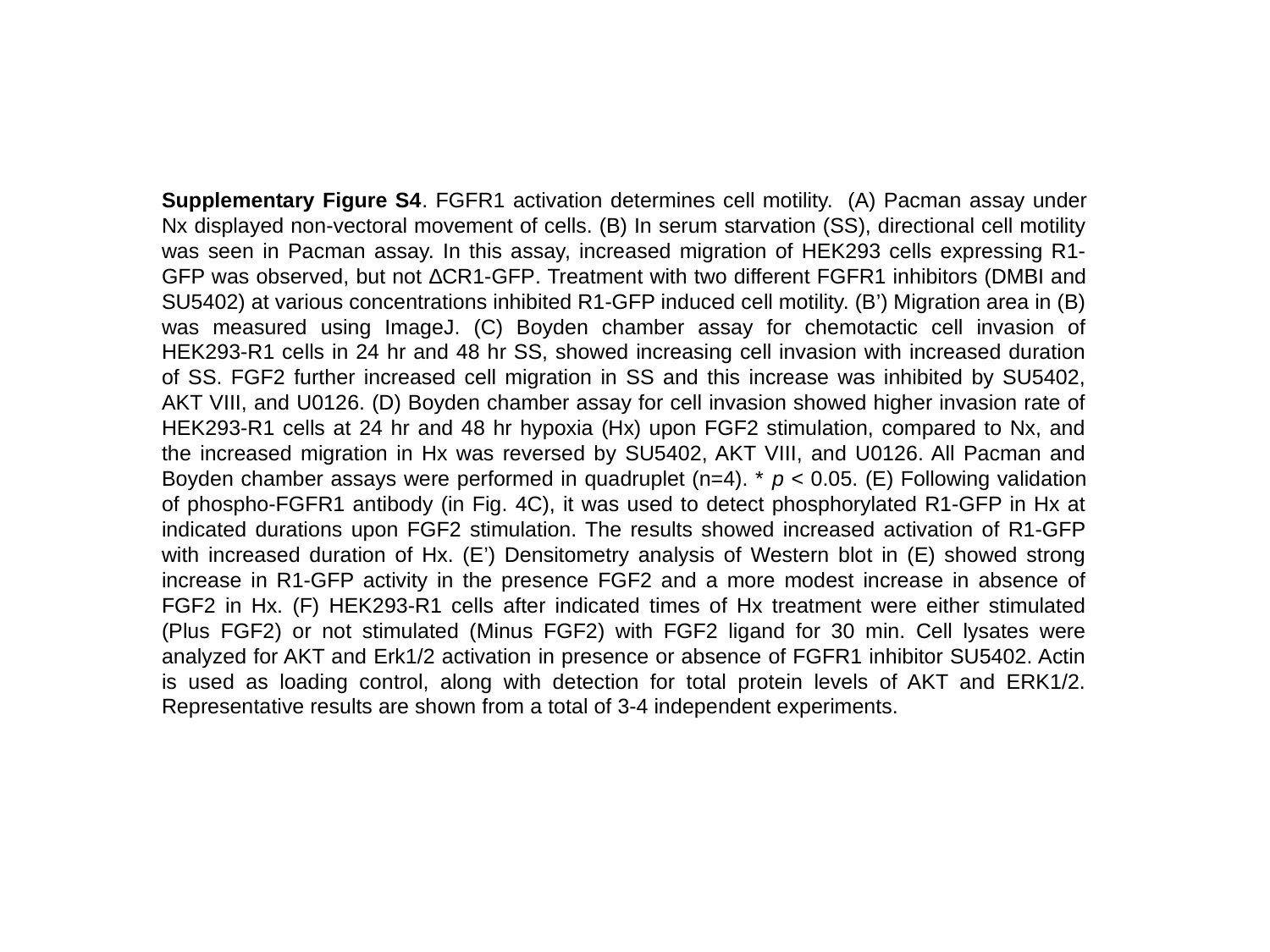

Supplementary Figure S4. FGFR1 activation determines cell motility. (A) Pacman assay under Nx displayed non-vectoral movement of cells. (B) In serum starvation (SS), directional cell motility was seen in Pacman assay. In this assay, increased migration of HEK293 cells expressing R1-GFP was observed, but not ∆CR1-GFP. Treatment with two different FGFR1 inhibitors (DMBI and SU5402) at various concentrations inhibited R1-GFP induced cell motility. (B’) Migration area in (B) was measured using ImageJ. (C) Boyden chamber assay for chemotactic cell invasion of HEK293-R1 cells in 24 hr and 48 hr SS, showed increasing cell invasion with increased duration of SS. FGF2 further increased cell migration in SS and this increase was inhibited by SU5402, AKT VIII, and U0126. (D) Boyden chamber assay for cell invasion showed higher invasion rate of HEK293-R1 cells at 24 hr and 48 hr hypoxia (Hx) upon FGF2 stimulation, compared to Nx, and the increased migration in Hx was reversed by SU5402, AKT VIII, and U0126. All Pacman and Boyden chamber assays were performed in quadruplet (n=4). * p < 0.05. (E) Following validation of phospho-FGFR1 antibody (in Fig. 4C), it was used to detect phosphorylated R1-GFP in Hx at indicated durations upon FGF2 stimulation. The results showed increased activation of R1-GFP with increased duration of Hx. (E’) Densitometry analysis of Western blot in (E) showed strong increase in R1-GFP activity in the presence FGF2 and a more modest increase in absence of FGF2 in Hx. (F) HEK293-R1 cells after indicated times of Hx treatment were either stimulated (Plus FGF2) or not stimulated (Minus FGF2) with FGF2 ligand for 30 min. Cell lysates were analyzed for AKT and Erk1/2 activation in presence or absence of FGFR1 inhibitor SU5402. Actin is used as loading control, along with detection for total protein levels of AKT and ERK1/2. Representative results are shown from a total of 3-4 independent experiments.

## Slide 6
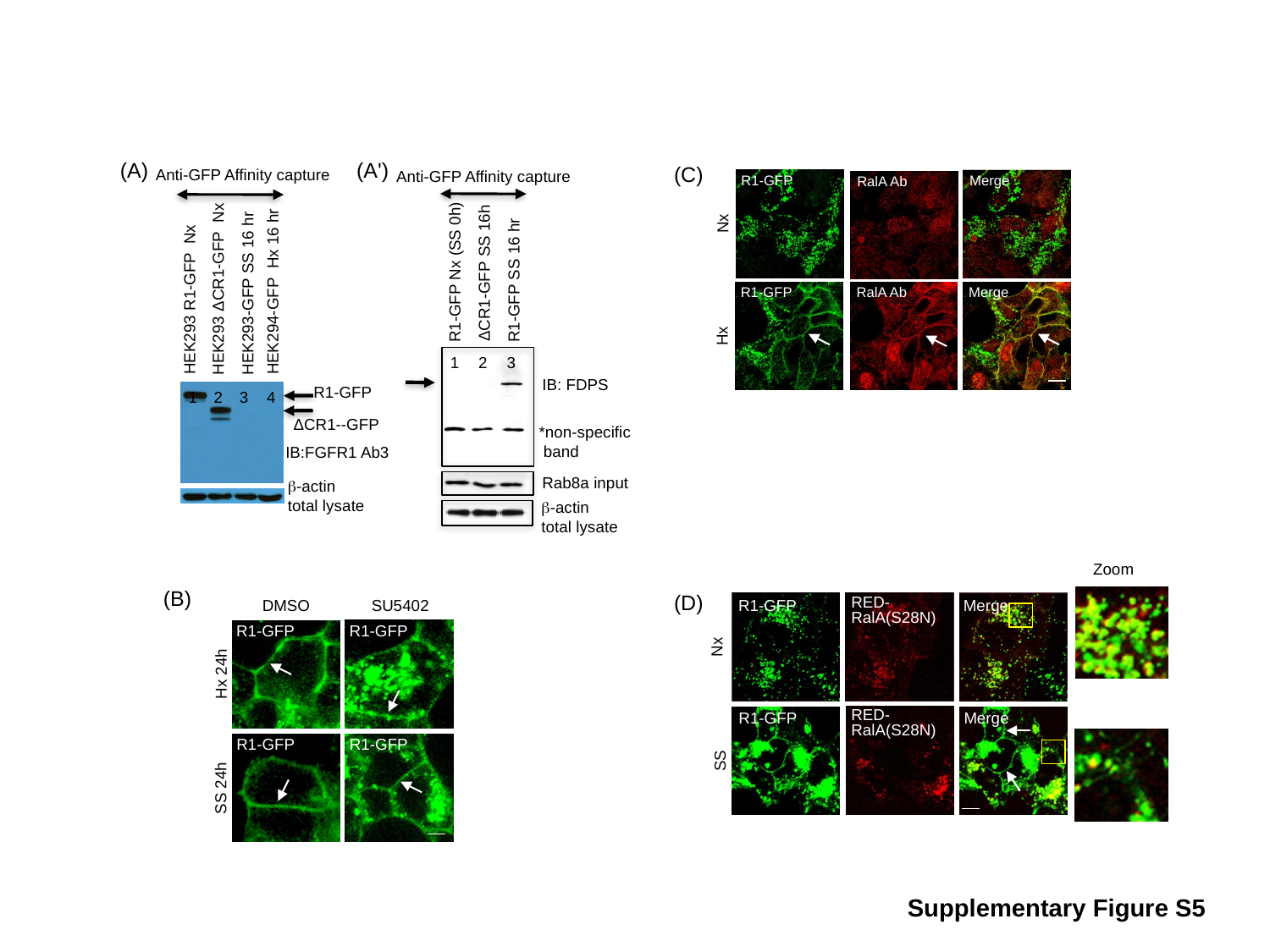

(A)
Anti-GFP Affinity capture
HEK293 ΔCR1-GFP Nx
HEK294-GFP Hx 16 hr
HEK293-GFP SS 16 hr
HEK293 R1-GFP Nx
R1-GFP
1
2
3
4
ΔCR1--GFP
IB:FGFR1 Ab3
b-actin
total lysate
(A')
Anti-GFP Affinity capture
R1-GFP Nx (SS 0h)
ΔCR1-GFP SS 16h
R1-GFP SS 16 hr
IB: FDPS
*non-specific
 band
Rab8a input
b-actin
total lysate
(C)
R1-GFP
Merge
RalA Ab
Nx
RalA Ab
Merge
R1-GFP
Hx
1
2
3
Zoom
(D)
R1-GFP
RED-
RalA(S28N)
Merge
Nx
R1-GFP
RED-RalA(S28N)
Merge
SS
(B)
DMSO
SU5402
R1-GFP
R1-GFP
Hx 24h
R1-GFP
R1-GFP
SS 24h
Supplementary Figure S5

## Slide 7
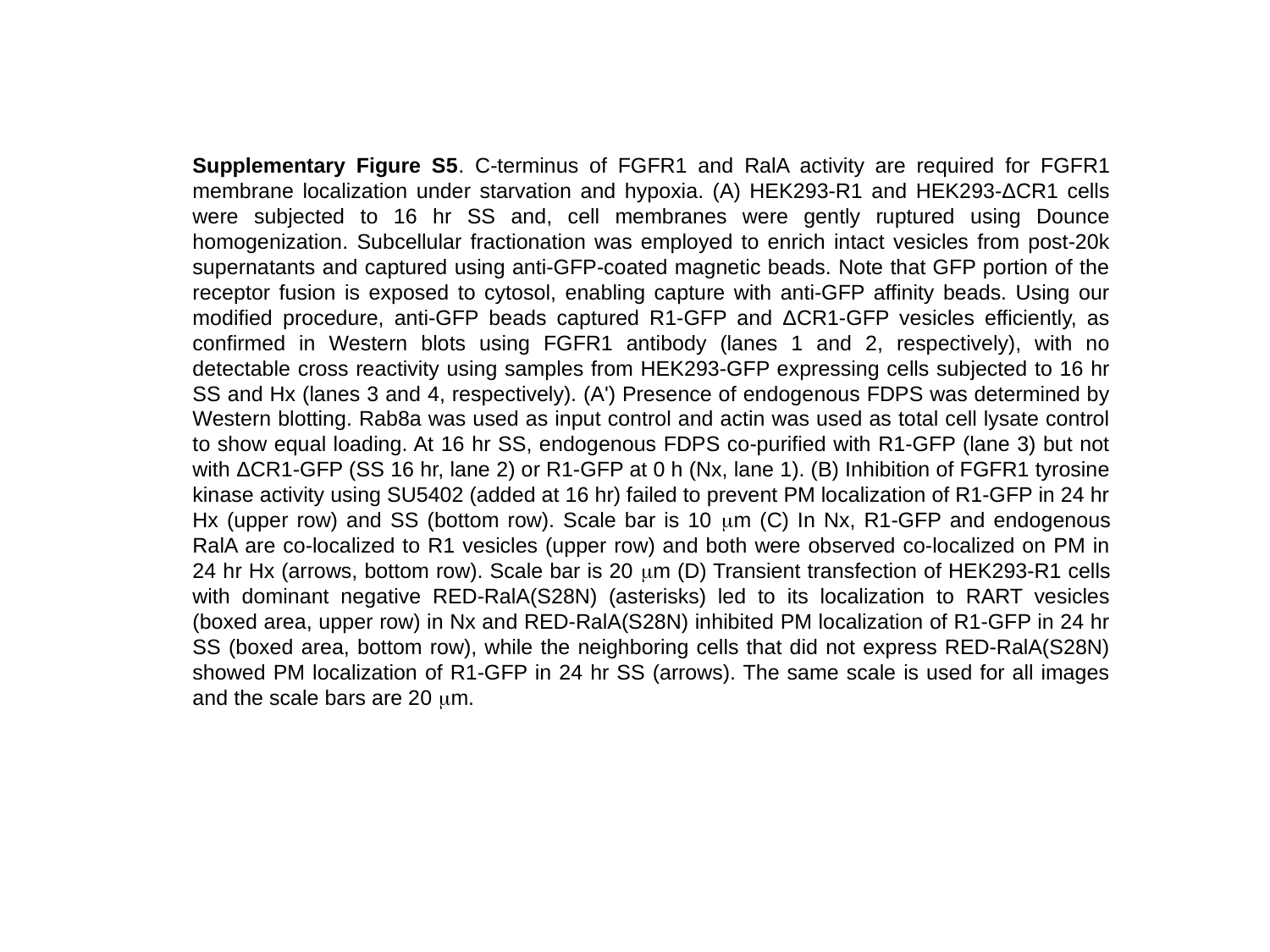

Supplementary Figure S5. C-terminus of FGFR1 and RalA activity are required for FGFR1 membrane localization under starvation and hypoxia. (A) HEK293-R1 and HEK293-ΔCR1 cells were subjected to 16 hr SS and, cell membranes were gently ruptured using Dounce homogenization. Subcellular fractionation was employed to enrich intact vesicles from post-20k supernatants and captured using anti-GFP-coated magnetic beads. Note that GFP portion of the receptor fusion is exposed to cytosol, enabling capture with anti-GFP affinity beads. Using our modified procedure, anti-GFP beads captured R1-GFP and ΔCR1-GFP vesicles efficiently, as confirmed in Western blots using FGFR1 antibody (lanes 1 and 2, respectively), with no detectable cross reactivity using samples from HEK293-GFP expressing cells subjected to 16 hr SS and Hx (lanes 3 and 4, respectively). (A') Presence of endogenous FDPS was determined by Western blotting. Rab8a was used as input control and actin was used as total cell lysate control to show equal loading. At 16 hr SS, endogenous FDPS co-purified with R1-GFP (lane 3) but not with ΔCR1-GFP (SS 16 hr, lane 2) or R1-GFP at 0 h (Nx, lane 1). (B) Inhibition of FGFR1 tyrosine kinase activity using SU5402 (added at 16 hr) failed to prevent PM localization of R1-GFP in 24 hr Hx (upper row) and SS (bottom row). Scale bar is 10 mm (C) In Nx, R1-GFP and endogenous RalA are co-localized to R1 vesicles (upper row) and both were observed co-localized on PM in 24 hr Hx (arrows, bottom row). Scale bar is 20 mm (D) Transient transfection of HEK293-R1 cells with dominant negative RED-RalA(S28N) (asterisks) led to its localization to RART vesicles (boxed area, upper row) in Nx and RED-RalA(S28N) inhibited PM localization of R1-GFP in 24 hr SS (boxed area, bottom row), while the neighboring cells that did not express RED-RalA(S28N) showed PM localization of R1-GFP in 24 hr SS (arrows). The same scale is used for all images and the scale bars are 20 mm.

## Slide 8
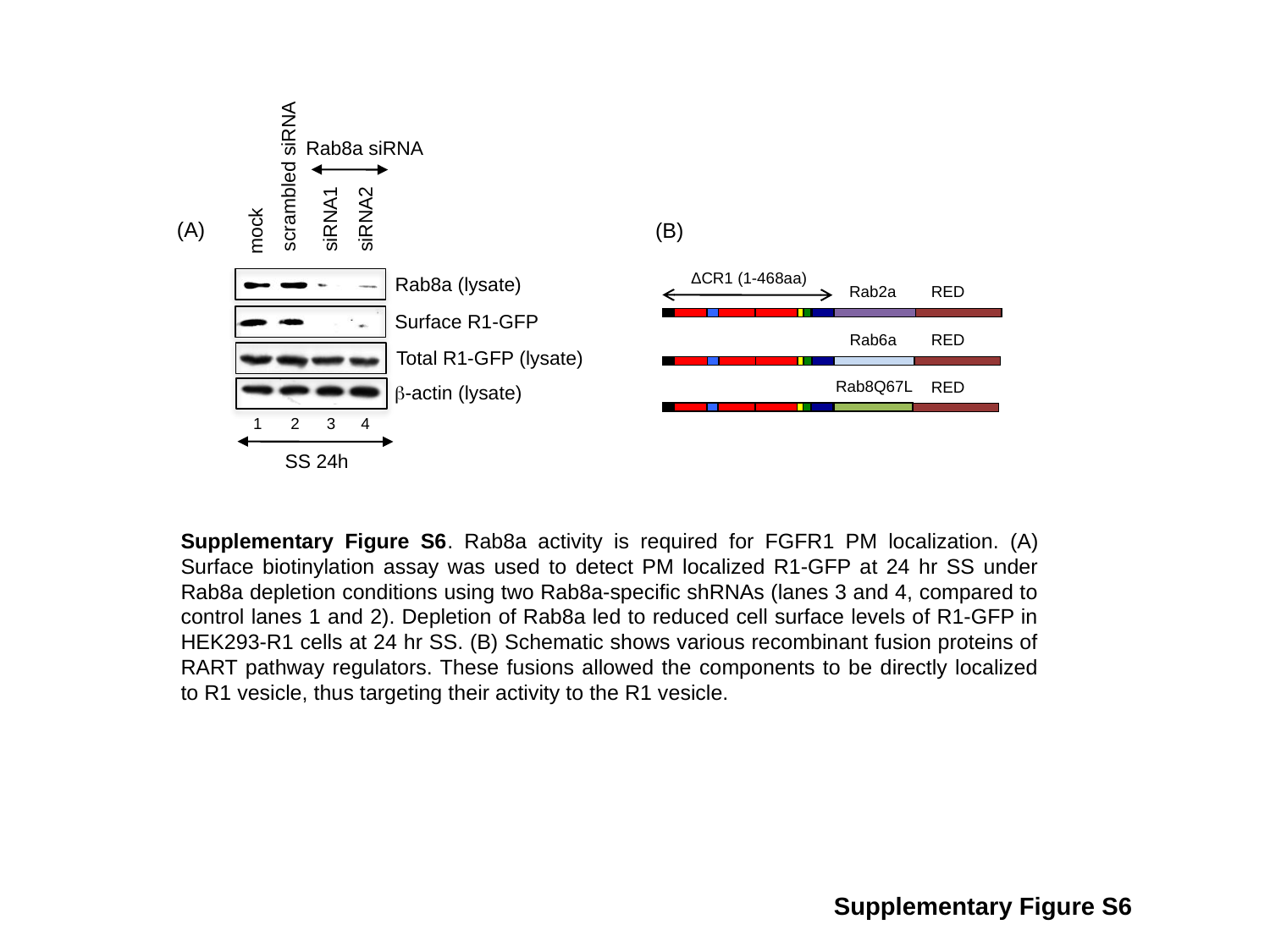

Rab8a siRNA
scrambled siRNA
siRNA2
siRNA1
(A)
(B)
mock
ΔCR1 (1-468aa)
Rab8a (lysate)
Rab2a
RED
Surface R1-GFP
RED
Rab6a
Total R1-GFP (lysate)
Rab8Q67L
RED
b-actin (lysate)
1
2
3
4
SS 24h
Supplementary Figure S6. Rab8a activity is required for FGFR1 PM localization. (A) Surface biotinylation assay was used to detect PM localized R1-GFP at 24 hr SS under Rab8a depletion conditions using two Rab8a-specific shRNAs (lanes 3 and 4, compared to control lanes 1 and 2). Depletion of Rab8a led to reduced cell surface levels of R1-GFP in HEK293-R1 cells at 24 hr SS. (B) Schematic shows various recombinant fusion proteins of RART pathway regulators. These fusions allowed the components to be directly localized to R1 vesicle, thus targeting their activity to the R1 vesicle.
Supplementary Figure S6

## Slide 9
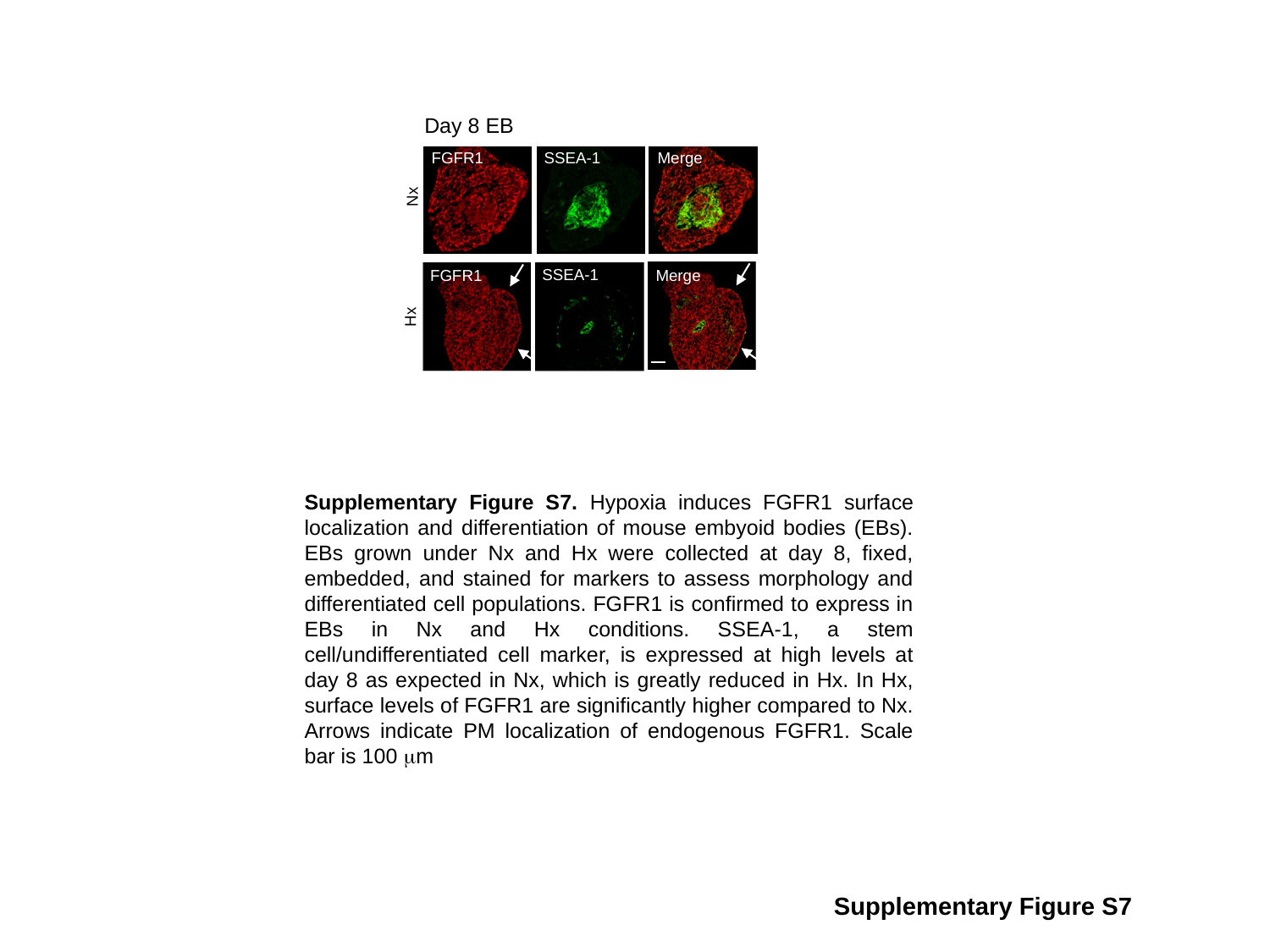

Day 8 EB
SSEA-1
FGFR1
Merge
Nx
SSEA-1
FGFR1
Merge
Hx
Supplementary Figure S7. Hypoxia induces FGFR1 surface localization and differentiation of mouse embyoid bodies (EBs). EBs grown under Nx and Hx were collected at day 8, fixed, embedded, and stained for markers to assess morphology and differentiated cell populations. FGFR1 is confirmed to express in EBs in Nx and Hx conditions. SSEA-1, a stem cell/undifferentiated cell marker, is expressed at high levels at day 8 as expected in Nx, which is greatly reduced in Hx. In Hx, surface levels of FGFR1 are significantly higher compared to Nx. Arrows indicate PM localization of endogenous FGFR1. Scale bar is 100 mm
Supplementary Figure S7
